# A light-driven dual-nanotransformer with deep tumor penetration for efficient chemo-immunotherapy

**DOI:** 10.7150/thno.68756

**Published:** 2022-01-24

**Authors:** Jiahui Peng, Fangman Chen, Yulu Liu, Fan Zhang, Lei Cao, Qiannan You, Dian Yang, Zhimin Chang, Mingfeng Ge, Li Li, Zheng Wang, Qian Mei, Dan Shao, Meiwan Chen, Wen-Fei Dong

**Affiliations:** 1 School of Biomedical Engineering (Suzhou), Division of Life Sciences and Medicine, University of Science and Technology of China, 96 Jinzhai Road, Hefei 230026, China.; 2 CAS Key Laboratory of Bio Medical Diagnostics, Suzhou Institute of Biomedical Engineering and Technology Chinese Academy of Sciences, 88 Keling Road, Suzhou 215163, China.; 3 Institutes of Life Sciences, School of Medicine, South China University of Technology, Guangzhou, Guangdong 510006, China.; 4 School of Biomedical Sciences and Engineering, South China University of Technology, Guangzhou International Campus, Guangzhou, Guangdong 511442, China.; 5 National Engineering Research Center for Tissue Restoration and Reconstruction, South China University of Technology, Guangzhou, Guangdong 510006, China.; 6 CAS Key Laboratory of Nano-Bio Interface, Suzhou Institute of Nano-Tech and Nano-Bionics, Chinese Academy of Sciences, 398 Ruoshui Road, Suzhou 215123, China.; 7 State Key Laboratory of Quality Research in Chinese Medicine, Institute of Chinese Medical Sciences, University of Macau, Macau, China.

**Keywords:** phototherapy, light response, tumor penetration, mesoporous organosilica nanoparticles, immunotherapy

## Abstract

Designing a transformable nanosystem with improved tumor accumulation and penetration by tuning multiple physicochemical properties remains a challenge. Here, a near-infrared (NIR) light-driven nanosystem with size and charge dual-transformation for deep tumor penetration is developed.

**Methods:** The core-shell nanotransformer is realized by integrating diselenide-bridged mesoporous organosilica nanoparticles as a reactive oxygen species (ROS)-responsive core with an indocyanine green (ICG)-hybrid N-isopropyl acrylamide layer as a thermosensitive shell. After loading doxorubicin (DOX), negatively charged nanomedicine prevents DOX leakage, rendering prolonged blood circulation time and high tumor accumulation.

**Results:** Upon NIR light irradiation, mild photothermal effects facilitate the dissociation of the thermosensitive shell to achieve negative-to-positive charge reversal. Meanwhile, ICG-generated ROS cleave the diselenide bond of the organosilica core, resulting in rapid matrix degradation that produces DOX-containing smaller fragments. Such a light-driven dual-transformable nanomedicine simultaneously promotes deep tumor penetration and implements sufficient chemotherapy, along with evoking robust immunogenic cell death effects *in vitro* and *in vivo*. With the combination of a programmed cell death protein-1 (PD-1) checkpoint blockade, the nanotransformer remarkably blocks primary tumor growth and pulmonary metastasis of breast cancer with low systemic toxicity.

**Conclusions:** This study develops a promising strategy to realize high tumor accumulation and deep penetration of light-transformable nanomedicine for efficient and safe chemo-immunotherapy.

## Introduction

In addition to killing tumor cells, chemotherapeutics, such as anthracyclines, taxanes, mitoxantrone, and oxaliplatin, induce immunogenic cell death (ICD), which sensitizes tumors to programmed cell death protein-1 (PD-1)/programmed cell death ligand-1 (PD-L1) blockade immunotherapy 1-3. Chemo-immunotherapy has proven to be a promising therapy for inhibiting the development and progression of solid tumors in preclinical studies and clinical trials 4-6. However, chemo-immunotherapy is primarily limited by off-target toxicity and the ineffectiveness of chemotherapeutics. To overcome these drawbacks, nanocarriers have been designed to achieve on-demand drug release in response to tumor microenvironments 7-11. Although nanomedicines improve the pharmacokinetics and ameliorate the adverse effects of free drugs, many of them fail to promote efficient outcomes in clinical trials due to insufficient tumor accumulation and penetration 12, 13. Biological barriers, characterized by a high degree of hypovascularity, dense extracellular matrix, and high interstitial fluid pressure in solid tumors, hinder the accumulation and deep penetration of nanocarriers 14-16. Therefore, the development of nanocarriers with deep tumor penetration is urgently required to achieve high-efficiency chemo-immunotherapy.

Blood circulation, tumor accumulation, and penetration of nanomedicines play important roles in cancer therapy 17, 18. It remains an unresolved obstacle for nanocarriers to increase blood circulation, promote tumor accumulation across tight blood endothelial cells, and facilitate deep penetration into distal tumor cells. The physicochemical properties of nanocarriers, including their size, shape, rigidity, charge, and surface chemistry, have profound effects on their pharmacokinetics and delivery efficiency 19, 20. Chan et al. proved that smaller PEGylated Au nanoparticles enhanced tumor accumulation and deep penetration via passive and active pathways 21. The positively charged nanocarriers show stronger interactions with the cell membrane, facilitating cellular uptake and transcytosis, and increasing their accumulation and penetration in avascular tumor sites 22, 23. There are multiple irreconcilable requirements for the physicochemical properties of nanocarriers at different delivery stages. Increasing evidence reveals that large size (100-200 nm) and negatively charged nanocarriers enable long blood circulation, but impair their tumor accumulation and deep penetration 23. To facilitate both accumulation and penetration of tumors, it is highly advantageous to develop sophisticated nanocarriers that can undergo physicochemical properties' transformation (e.g., large to small size, negative to positive charge) in response to multiple stimuli, such as reactive oxygen species (ROS), pH, enzymes, and external stimuli 24-26.

Smart nanomedicines with high penetration-promoting properties can be classified as size-shrinkable nanoparticles, “cluster bomb”-like sequential-releasing nanoparticles, charge-reversal nanoparticles, and penetrating peptide-modified nanoparticles 27. For instance, Shen et al. devoted considerable effort to producing charge-transformable nanocarriers in response to special enzymes with high expression levels in tumor tissue, achieving high accumulation and deep tumor penetration via transcytosis effects across vascular endothelial cells and tumor cells 24, 28. However, endogenous stimuli affected by tumor heterogeneity might reduce the responsiveness of the nanocarriers, impeding their further application. Exogenous stimuli, such as: light, radiation, heat, and electric and magnetic fields, are more controllable and homogeneous 27, 29-31. Among them, NIR light in the “therapeutic window” (720-900 nm) is considered to be one of the most promising stimuli because of its advantages of: spatiotemporal control, deep tissue penetration, non-invasion, and safety 27, 31. Nevertheless, most of these transformable nanocarriers are triggered by a single stimulus. Therefore, the design of an NIR light-driven nanocarrier with multiple transformable capabilities remains a challenge, which could further facilitate tumor accumulation and penetration.

**Scheme 1:**
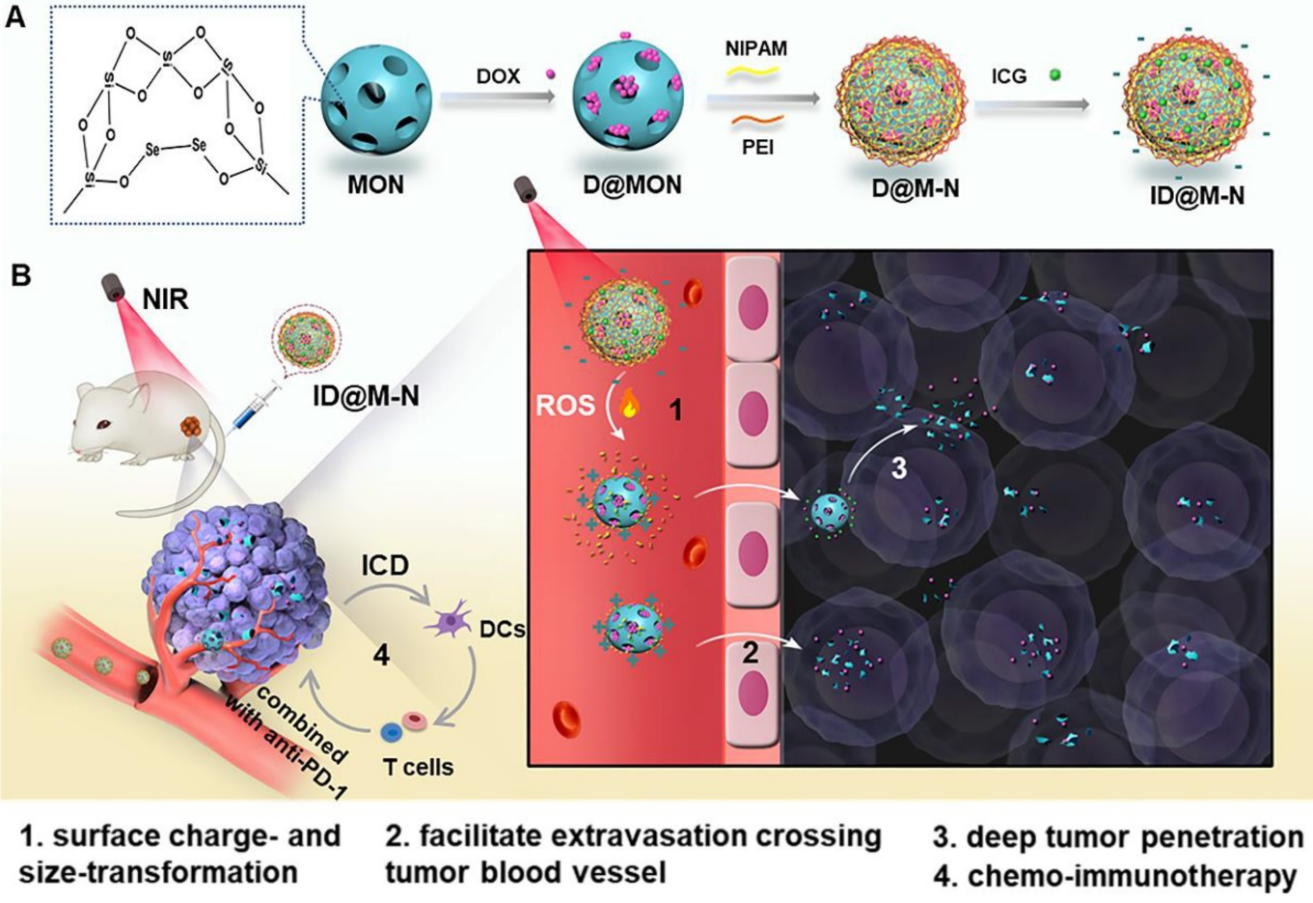
**(A)** Schematic preparation of the core-shell dual-nanotransformer (ID@M-N). **(B)** NIR light triggered size- and charge-transformability for enhanced chemo-immunotherapy. The ID@M-N is disassociation due to photothermal effects, resulting in charge reversal from negative to positive. Simultaneously, ROS triggered degradation into smaller segments. The transformability facilitated accumulation and deep tumor penetration.

Herein, we developed a size and charge dual-nanotransformer (ID@M-N) for enhanced chemo-immunotherapy. As illustrated in Scheme 1, diselenide-bridged mesoporous organosilica nanoparticles were designed as a ROS-responsive core for the chemotherapeutic agent DOX loading, and the ICG hybrid N-isopropyl acrylamide (NIPAM) layer was then coated to form a thermosensitive shell. Negatively charged ID@M-N exhibited prolonged blood circulation and prevented DOX leakage. Under NIR light irradiation, the dissociation of the shell is realized by mild photothermal effects, resulting in a surface charge-reversal, from negative to positive, at the tumor site. Meanwhile, the dissociated core is further cleaved into smaller fragments by ICG-produced ROS, which converts the particulate size from 115 to 20 nm. Such an NIR light-driven nanotransformer facilitates tumor accumulation and deep penetration, amplifies chemotherapy potency, and evokes robust ICD effects *in vitro* and* in vivo*. With the help of the PD-1 checkpoint blockade, dual-nanotransformers not only inhibited primary tumor growth in an efficient and safe manner, but also exhibited great anti-metastatic efficacy in 4T1 orthotopic mammary tumor-bearing mice.

## Materials and Methods

### Materials

Tetraethyl orthosilicate (TEOS), etyltrimethylammonium tosylate (CTAT), triethanolamine (TEA), 3-aminopropyltriethoxysilane (APTES) and fluorescein isothiocyanate (FITC) were purchased from Sigma-Aldrich Co. (St Louis, MO, USA). Ammonium nitrate (NH_4_NO_3_) and hydrogen peroxide (30% H_2_O_2_) were purchased from Beijing Chemical Reagent Co. (Beijing China). Methacryloxy propyl trimethoxyl silane (MPS), ammonium persulphate (APS), *N, N'*-Methylenebisacrylamide (MBA), *N*-Isopropyl acrylamide (NIPAM), L (+)-Ascorbic acid (Vitamin C) and Indocyanine green (ICG) were purchased from Shanghai Titan Scientific Co., Ltd. (Shanghai China). Polyethyleneimine (PEI), branched (average Mw~25,000 by LS) was purchased from Aldrich Chemical Co (Darmstadt, Germany). *3*-(Trimethoxysilyl) propyl Methacrylate (MPS) was purchased from Tokyo Chemical Industry Co., LTD (Tokyo, Japan). Doxorubicin hydrochloride (DOX) and *1,3*-diphenylisobenzofuran (DPBF) were purchased from Energy Chemical Technology Co., LTD (Shanghai China). Fetal bovine serum (FBS), 0.25% Trysin-EDTA, RPMI Medium Modified (1640) and penicillin-streptomycin antibiotics were purchased from Gibco Co., Ltd. (Carlsbad, CA, USA). WST-1 cell proliferation and cytotoxicity test kit (WST-1), fluoroshield Mounting Medium with DAPI, 4% paraformaldehyde, ATP Assay Kit, FITC-labeled Goat Anti-Rabbit IgG, HSP70 rabbit monoclonal antibody and anti-CD31 antibody, were purchased from Beyotime Biotechnology (Shanghai, China). Reactive oxygen species detection kit was purchased from Solarbio (Beijing, China). Rabbit anti-calreticulin antibody (ab223614) was purchased from Abcam (Shanghai, China). Anti-mouse CD11c-FITC, anti-mouse CD80-PE, anti-mouse CD86-APC, mouse TNF-α ELISA Kit, IFN-γ ELISA Kit, anti-CD4-PE and anti-CD8a-APC were purchased from Multi Sciences Biotech Co., Ltd. (Hangzhou, China). HMGB1 ELISA kit was purchased from Jingmei Biotech Co., Ltd. (Yancheng, China). Anti-CD3-FITC was purchased from Thermo Fisher Scientific (USA).

### Synthesis of biodegradable MONs

A selenium-containing silicon source, *bis*[*3*-(triethoxysilyl) propyl]diselenide (BTESePD), which is required for the synthesis of degradable MONs, was prepared in the laboratory as previously described 32. The MONs were synthesized using the following modified sol-gel method: CTAT (0.6 g) and TEA (0.15 g) were dispersed in deionized water (40 mL), stirred for 30 min at 80 °C, following which, a mixture of TEOS (4 g), BTESePD (1 g) and ethanol (1 mL) was added dropwise. After stirring continuously for 4 h, the products were collected by centrifugation, washed three times with ethanol, and refluxed for 12 h in an ethanol solution of NH_4_NO_3_ (1% w/v). Finally, the MONs were collected, washed, vacuum-dried, and stored at 4 °C. For DOX loading and NIPAM layer coating, we modified the carboxylate groups on the surface and pores of MON, as described previously 32.

### Synthesis of thermosensitive MON-NIPAM (M-N)

The obtained MON was resuspended in deionized water (15 mL) and mixed with ethanol solution (15 mL) containing PEI (50 mg), NIPAM (0.24 g), and MBA (40 mg). After 4 h, nitrogen was continuously poured into the mixed solution for deoxygenation, an appropriate amount of APS (1-2 mg) was added, and the mixture was stirred for 12 h under nitrogen at 30 °C. The reaction products were collected and washed. Finally, the yellow powder was dried under a vacuum for 8 h at 30 °C and stored at 4 °C. For the synthesis of FITC-labeled NIPAM (M-N-FITC), M-N was mixed with 1% (w%) FITC in aqueous solution and stirred overnight at room temperature, after which M-N-FITC was collected and washed with water three times for subsequent experiments.

The drug-loaded nanoparticles (NPs) are prepared as follows: DOX (2.5 mg, 0.5 mg mL^-1^) was added into MON (10 mg, 2 mg mL^-1^) solution. After stirring for 4 h, the reaction solution was centrifuged to obtain DOX@MON (D@MON). D@MON was used to coat the thermosensitive layer. The ICG@DOX@MON-NIPAM (ID@M-N) was prepared as follows: the previously obtained D@M-N (10 mg, 2 mg mL^-1^) was mixed with ICG (5 mg) and stirred, in the dark, for 4 h at room temperature. The reaction solution was then centrifuged to collect ID@M-N. For the preparation of ICG@MON-NIPAM (I@M-N), the same method was adopted without the use of DOX. To characterize drug co-loading, the UV-vis spectra of M-N, free DOX, free ICG, and ID@M-N were recorded using an ultraviolet spectrophotometer (Hitachi, U-3900H, Japan). The UV absorption value (DOX:480 nm, ICG:780 nm) of the supernatant was determined after centrifugation using a multimode reader (Bio-Tek Synergy HT, USA). Briefly, encapsulation efficiency (EE%) and drug loading capacity (LC%) were calculated as follows:

EE % = 100 % × Amount of drug in NPs/Total amount of drug added (1);LC % = 100 % × Amount of drug in NPs/NPs weight (2)

### Measure of the lower critical solution temperature (LCST)

First, D@M-N (0.5 mg) was dispersed in deionized water (6 mL) and incubated at 120 rpm; and the amount of drug released at 35 °C, 40 °C, 45 °C, 50 °C, 55 °C, and 60 °C was measured for 1 h to determine the LCST of the thermosensitive layer.

### Characterization and biodegradation of MONs

Fourier transform infrared (FTIR) spectra of MONs, and MON-NIPAM were recorded to determine whether the thermosensitive layer was successfully prepared using a Bruker model VECTOR22 Fourier transform spectrometer. The pore size distribution and specific surface area of MON and MON-NIPAM were evaluated and calculated using the Brunauer-Emmett-Teller (BET) and Barrett-Joyner-Halenda (BJH) methods using a BSD-PS2 surface area porosity analyzer (BEISHIDE Instrument Technology Co., Ltd, Beijing, China). The morphologies of MON and MON-NIPAM were observed using transmission electron microscopy (TEM, JEM-1400 plus, Japan) and scanning electron microscopy (SEM, FEI Quanta FEG250). Energy dispersive X-ray Spectroscopy (EDS) of MON was performed with a JEM-2100F EDX system. The hydration particle size distribution and surface zeta potential of different preparations were measured using a Malvern Nano-ZS90 (DLS, Malvern Instruments Ltd., Worcestershire, UK) at room temperature. The proportion of the thermosensitive layer to the weight of MON-NIPAM was determined using an STA 449F3 Jupiter synchronous thermal analyzer (TGA, NETZSCH-Gerätebau GmbH, Germany). The variation in the particle size distribution of NPs over time in 50% FBS at 37 °C was measured by DLS as an indicator of *in vivo* stability. The UV-vis absorption spectra of I@M-N and free ICG were recorded over time at 4 °C on an ultraviolet spectrophotometer (U-3900H, Japan) to determine the stability of the drugs. The biodegradability of the diselenide-bridged MON was characterized by its morphology. Briefly, MON (100 μg mL^-1^) was dispersed in aqueous solutions of H_2_O_2_ (100 μM) and then incubated at 37 °C to simulate the intracellular conditions, and samples were collected at predetermined time points (1 and 3 days) for TEM examination.

### Evaluation of PTT and PDT efficacy* in vitro*

The temperature changes of PBS, free ICG and ID@M-N (0.25 W cm^-1^, ICG: 20 μg mL^-1^) were recorded using a 225S online thermal imaging camera (FOTRIC, China) for 10 min. *1,3*-Diphenylisobenzofuran (DPBF) was used to evaluate the PDT efficacy of preparations, and vitamin C (Vc) was used as a ROS scavenger. Briefly, free ICG (0.5 mL) and ID@M-N (ICG: 10 μg mL^-1^) with or without Vc were rapidly mixed with DPBF (0.5 mL). After irradiation with NIR light (0.25 W cm^-2^, 8 min), the UV-vis absorption spectra of the different solutions were recorded, and PBS was used as a blank control.

### DOX release behavior *in vitro*

The NIR stimuli-responsive behavior and the effects of simulated intracellular redox conditions were investigated by dispersing ID@M-N (0.5 mg) in deionized water (20 mL) containing H_2_O_2_ (100 μM) and incubated at 120 rpm and 37 °C. Among them, the H_2_O_2_ and H_2_O groups were initially either exposed or not exposed to irradiation (0.25 W cm^-2^, 10 min). The amount of DOX in supernatant was quantified at predetermined time points using UV-vis measurements.

### Cellular uptake behavior

4T1 cells were cultured in RPMI-1640 medium supplemented with 1% penicillin-streptomycin antibiotics and 10% FBS in an incubator with a humidified atmosphere containing 5% CO_2_ at 37 °C. To investigate the effect of NIR light on the uptake of different nanoparticles, 4T1 cells were seeded into confocal glass dishes (20 mm) with 100,000 cells and cultured overnight. FITC-labeled ID@M-N (DOX: 10 μg mL^-1^) was pretreated with 808 nm light for 10 min (0.25 W cm^-2^) and was then incubated with cells for 0.5, 1, and 2 h. After fixation with 4% paraformaldehyde, the cell nuclei were stained with DAPI for observation using a confocal laser scanning microscope (CLSM, Leica TCS SP8, Germany).

### Transport across blood endothelial cells

A Transwell system (Corning 3422) was used to study the transport of ID@M-N into endothelial cells. First, HUVECs were incubated for 3 days in the upper compartment at a density of 500,000 cell mL^-1^ to promote the formation of a dense layer of cells, and 4T1 cells (200,000 cell mL^-1^) were seeded into the bottom compartment for 24 h. ID@M-N with an FITC-labeled core was added to the upper compartment and exposed to NIR light (0.25 W cm^-2^, 10 min). After culturing for 24 h, the MFI of FITC in the medium and 4T1 cells was detected using a microplate reader and flow cytometry, respectively.

### Penetration of tumor multicellular spheroids (MCSs)

Tumor multicellular spheroids (MCSs) were established. Briefly, 4T1 cells (4×10^-5^ per well) mixed with matrix gel (50 μL) were added to 96-well plates pretreated with 1% agarose (50 μL) per well. After growing to the appropriate size, MCSs were co-incubated with FITC-labeled ID@M-N upon light irradiation (0.25 W cm^-2^, 10 min). After culturing for 4 h, the MCSs were washed three times with PBS. Penetration of nanoparticles in MCSs was observed using CLSM.

### Intracellular ROS production and NIR drive core-shell dissociation

4T1 cells (100,000 cells) were cultured in confocal glass dishes overnight. The cells were then incubated with ID@M-N and I@M-N for 2 h. After light irradiation (0.25 W cm^-2^, 10 min), cells were incubated with DCFH-DA for 1 h at 37 °C, washed, fixed, and stained with DAPI for observation by CLSM.

To further investigate the ROS-mediated core-shell dissociation of the nanotransformer, FITC-labeled ID@M-N (2 μg mL^-1^) was incubated with 4T1 cells as described above. After incubating for 2 h, the cells were either irradiated or not irradiated (0.25 W cm^-2^, 10 min). After culturing for another 4 h, the cells were stained with DAPI, and the fluorescent signals of FITC and DOX were observed by CLSM.

## Results and Discussion

### Synthesis and physicochemical characterization of ID@M-N

**Figure 1:**
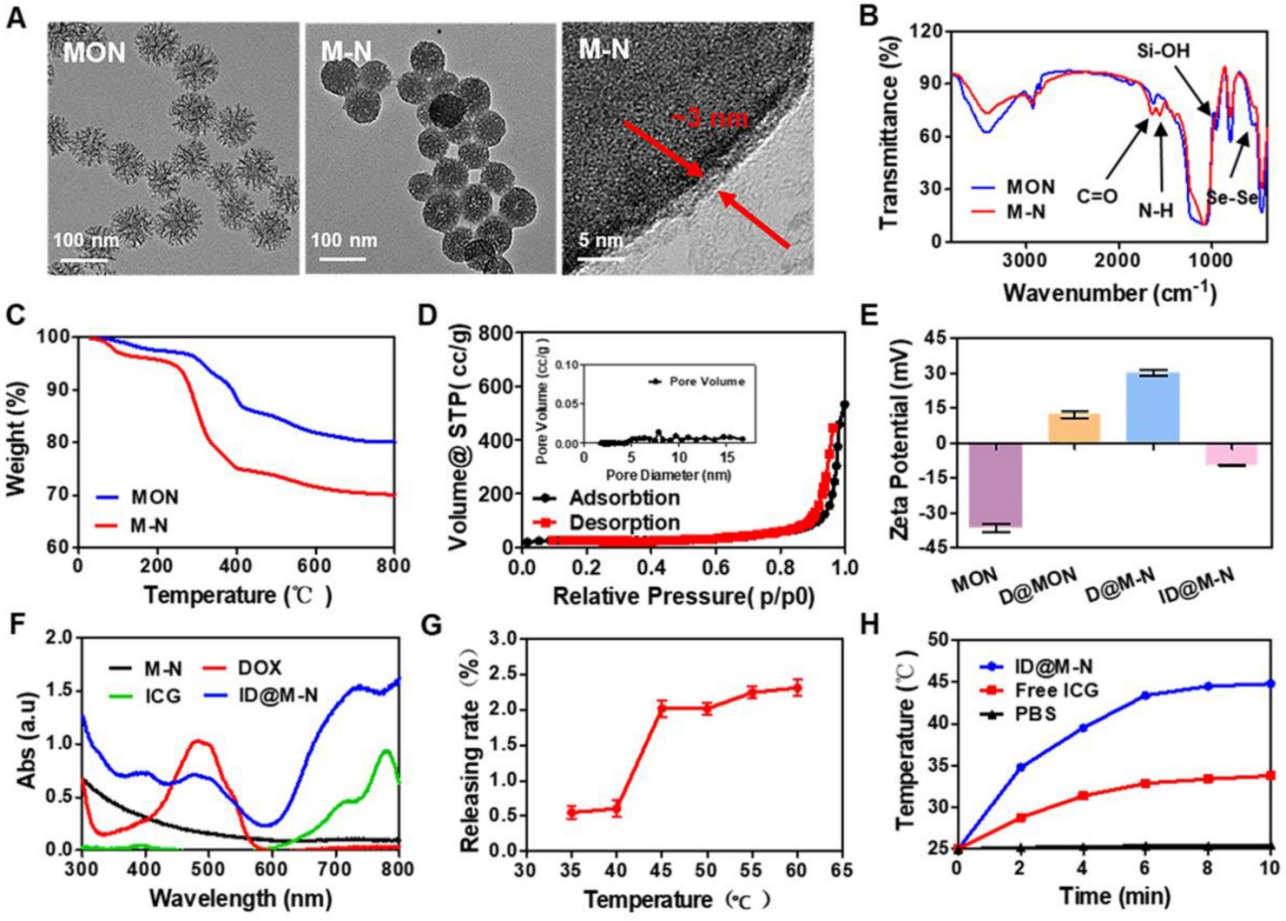
** Preparation and characterization of nanotransformer. (A)** TEM images of MON, M-N, and the magnified temperature sensitive layer. **(B)** FTIR spectra and, **(C)** thermogravimetric analysis (TGA) of MON and M-N. **(D)** Nitrogen adsorption-desorption isotherms of M-N. **(E)** Zeta potential of MON, D@MON and D@M-N, and ID@M-N. **(F)** UV-vis spectra of M-N, free DOX, free ICG and ID@M-N. **(G)** Lower critical solution temperature (LCST) of ID@M-N. **(H)** Photothermal profiles of ID@M-N upon 808 nm light irradiation (ICG: 20 µg mL^-1^, 0.25 W cm^-2^).

Mesoporous organosilica nanoparticles (MONs) have been widely employed in the delivery of chemotherapeutics because of their controllable physicochemical properties and degradation behavior in response to multiple stimuli 33-36. Diselenide-bond-bridged MONs with a high content of Se as the core of ID@M-N were fabricated using a modified sol-gel method 33. MONs exhibited a uniformly spherical morphology with a diameter of approximately 80 nm (Figure 1A, Figure S1). EDS mapping analysis revealed that Se element was homogeneous distribution in MON nanoparticles (Figure S2), and the content of Se element was account for 10.9 % determining by inductive coupled plasma emission spectrometer (ICP-OES). Thermosensitive NIPAM mixed with cationic polyethyleneimine (PEI) was coated on the surface of the MONs to obtain M-N using N, N'-methylenebisacrylamide (MBA) as the cross-linking agent and ammonium persulphate (APS) as the initiator 37, 38. After coating with the thermosensitive polymer, the surface of the M-N nanoparticles was smoother than that of the MONs (Figure 1A). High-resolution TEM images further showed that the thermosensitive layer covered on surface of MON to form a core-shell structure, and thickness of the thermosensitive layer to be about 3 nm. DLS results also confirmed that size increased after coating thermosensitive layer (Figure S3). Additionally, as shown in the FTIR spectra (Figure 1B), the peaks of the Se-Se and Se-C bonds at 550-750 cm^-1^, while the characteristic peaks observed at 1650 cm^-1^ (C=O stretching) and 1553 cm^-1^ (N-H stretching) belong to the amide bands of the thermosensitive polymer, further confirming the effective modification of the thermosensitive layer on the surface of the MONs. TGA results indicated that the thermosensitive layer coating on the surface of M-N accounted for approximately 10 wt% (Figure 1C). The N_2_ adsorption-desorption isotherms were recorded, and the pore size distributions of the nanoparticles were determined (Figure 1D, Figure S4, Table S1). The mesoporous structures of MONs and M-N showed typical type-IV isotherms. The BET surface area, total pore volume and average pore size decreased from 541.07 m^2^ g^-1^, 1.01 cm^3^ g^-1^, and 8.16 nm to 78.69 m^2^ g^-1^, 0.77 cm^3^ g^-1^, and 2.69 nm, respectively. The thermosensitive layer might be considered as the “gatekeeper” as it blocks the pore channels of MONs.

To prepare D@M-N, the MON loaded with DOX (Figure S1) was used to coat the thermosensitive layer. ICG was mixed with D@M-N to obtain ID@M-N at 25 °C while in the dark. The corresponding reversal charge and characteristic UV-vis absorbance spectra of ID@M-N (DOX: 480 nm; ICG: 780 nm) confirmed that DOX and ICG were encapsulated (Figure 1E-F). The loading capacity (LC) of DOX and ICG was 22.90% and 11.96%, respectively, as determined by the standard curve based on the UV-vis spectra (Figure S5). The LCST of the thermosensitive layer was established. The degree of DOX release from ID@M-N showed an obvious mutation at 40-45 °C, indicating the LCST (Figure 1G). The LCST was higher than the body temperature of the mice to effectively prevent DOX leakage. In addition, ID@M-N exhibited excellent colloidal stability over 7 days in culture media containing 50% serum (Figure S6), which may facilitate long blood circulation and reduce the clearance of the reticuloendothelial system.

The photobleaching of ICG usually hinders its application 39. The ICG in ID@M-N was more stable than free ICG for more than a week (Figure S7). To avoid photodamage of normal tissue around the tumor site, photothermal properties were further examined upon irradiation with 808 nm light (0.25 W cm^-2^, 10 min). ID@M-N exhibited a mild photothermal effect (~44 °C) (Figure 1H). This is higher than the LCST of ID@M-N.

### *In vitro* dual-transformation of charge and size by NIR irradiation

**Figure 2:**
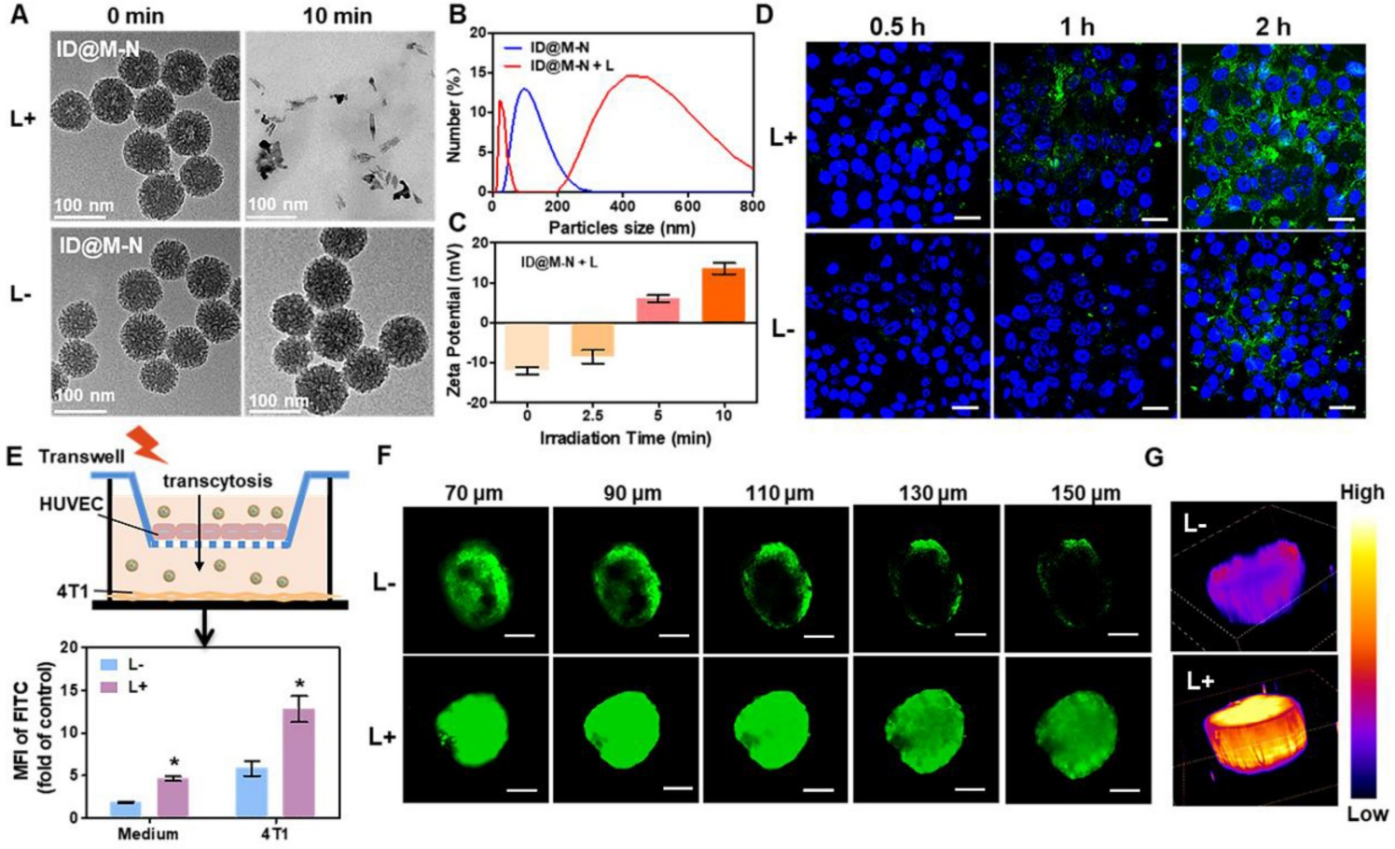
** NIR light drive charge- and size-transformation facilitating tumor penetration *in vitro*. (A)** TEM images and **(B)** DLS showing change in particle size of ID@M-N after light irradiation (10 min, 0.25 W cm^-2^). **(C)** Zeta potential of ID@M-N at different times of light irradiation **(D)** 4T1 cells uptake of ID@M-N with or without NIR light pretreated (10 min, 0.25 W cm^-2^). Blue fluorescence: nucleus; green fluorescence: FITC-labeled-ID@M-N; scale bars: 30 µm.** (E)** Transwell model for investigating vascular permeability of ID@M-N under NIR light irradiation (10 min, 0.25 W cm^-2^). The MFI of FITC from ID@M-N in the medium and 4T1 tumor cells cross the HUVEC blood vessel with or without light irradiation. ^*^ P < 0.05 compared with the corresponding (L-) group. **(F)** Tumor penetration of ID@M-N in MCSs observed by Z-stack CLSM. Scale bars: 100 µm. **(G)** 3D fluorescence analysis of CLSM at 150 µm depth. All data are mean ± SD (n = 3).

The NIR light-driven dual-transformation of charge and size was systematically investigated. Upon irradiation with NIR light, the ICG loading thermosensitive layer was dissociated, and the inner MONs rapidly degraded into smaller fragments (Figure 2A). In contrast, in the absence of NIR light, the core-shell ID@M-N nanotransformer remained integrated. The hydrodynamic diameter of ID@M-N shifted from a single distribution at approximately 115 nm to a dual distribution at 405 and 20 nm upon light irradiation (Figure 2B). The smaller sized particles were degradable fragments, and the larger particles could be attributed to the dissociation of the shell and aggregation of degradable fragments. As shown in Figure 2C, the initial negative charge of ID@M-N (-12.03 ± 0.51 mV) was gradually reversed to a positive charge (+13.5 ± 0.62 mV), close to that of D@MON (+14.6 ± 0.87mV). The results revealed the dissociation of the thermosensitive layer upon light irradiation. To further explore the light-driven transformation mechanism, the production of singlet oxygen (^1^O_2_) by ICG under NIR light irradiation was determined (Figure S8). We speculated that singlet oxygen (^1^O_2_), a key species of ROS 40, cleaved the diselenide bond-bridged matrix of ID@M-N, and triggered the dissociation and degradation, resulting in dual-transformation of the surface charge and size. Vitamin C (Vc), an ^1^O_2_ scavenger 41, markedly attenuated ^1^O_2_ production and decelerated the degradation of ID@M-N upon light irradiation (Figure S9), further verifying our hypothesis. Together, these results revealed that ROS and heat from the ID@M-N-based photothermal process facilitated the transformation of both charge and size.

### *In vitro* tumor penetration

To evaluate cellular uptake, FITC-labeled ID@M-N was pretreated with light irradiation for 10 min before incubation with 4T1 cells. As shown in Figure 2D, the uptake of ID@M-N was time-dependent. The fluorescence intensity of the 4T1 cells treated with light was stronger than that of ID@M-N without irradiation. Furthermore, quantification analysis confirmed that 4T1 cell uptake of ID@M-N pretreated with light was 2.2-fold higher than that of ID@M-N after incubation for 2 h (Figure S10). The cellular uptake of the transformable ID@M-N was enhanced by NIR light irradiation.

The tumor blood vessel was the first biological barrier to hinder extravasation, while the positive surface charge of the nanocarrier could facilitate extravasation crossing the tumor blood vessel 42. To determine the extravasation crossing vessel, the NIR light-driven transformation of ID@M-N was investigated using the Transwell model (Figure 2E). The vascular endothelial cells (HUVECs) were cultured in the upper chamber, and 4T1 tumor cells were cultured at the bottom of the basal chamber. The integral HUVEC barrier was constructed according to previous methods 43. After HUVECs were incubated with the FITC-labeled ID@M-N upon light irradiation, the mean fluorescence intensity (MFI) of FITC in the bottom of the medium and tumor cells was measured to evaluate their vascular permeability *in vitro*. The MFI of FITC-labeled ID@M-N with NIR light irradiation in the medium was 2.4 times higher than that of its counterpart. The results revealed that NIR light-driven ID@M-N enhanced transcellular transport across the HUVEC cell barrier. After crossing the HUVEC cell layer, the positive fragments of ID@M-N were rapidly internalized by 4T1 cells. The cellular fluorescence of FITC in light-exposed ID@M-N was 2.2 times higher than that of ID@M-N.

The 4T1 cell-derived multicellular spheroids (MCSs) were further used as three-dimensional models to mimic the morphology and biological microenvironment of solid tumors *in vitro*. FITC-labeled ID@M-N was used to evaluate penetration by scanning confocal laser scanning microscopy (CLSM) using a Z-stack at 20-μm intervals. The FITC-labeled ID@M-N only appeared in the superficial layer of the MCSs without NIR light irradiation (Figure 2F). In comparison, FITC-labeled ID@M-N was evenly homogeneous distributed of the whole MCSs after light irradiation (0.25 W cm^-2^, 10 min). As shown in Figure 2G, the 3D fluorescence imaging showed that the fluorescence of FITC-labeled ID@M-N with light irradiation inside the MCSs was clearly observed at a depth of 150 μm, whereas the fluorescence was very weak without light irradiation at the same depth. These results collectively proved that the light-driven transformable ID@M-N achieved deep penetration in MCSs.

### *In vitro* drugs release and light-responsive Chemotherapy

**Figure 3:**
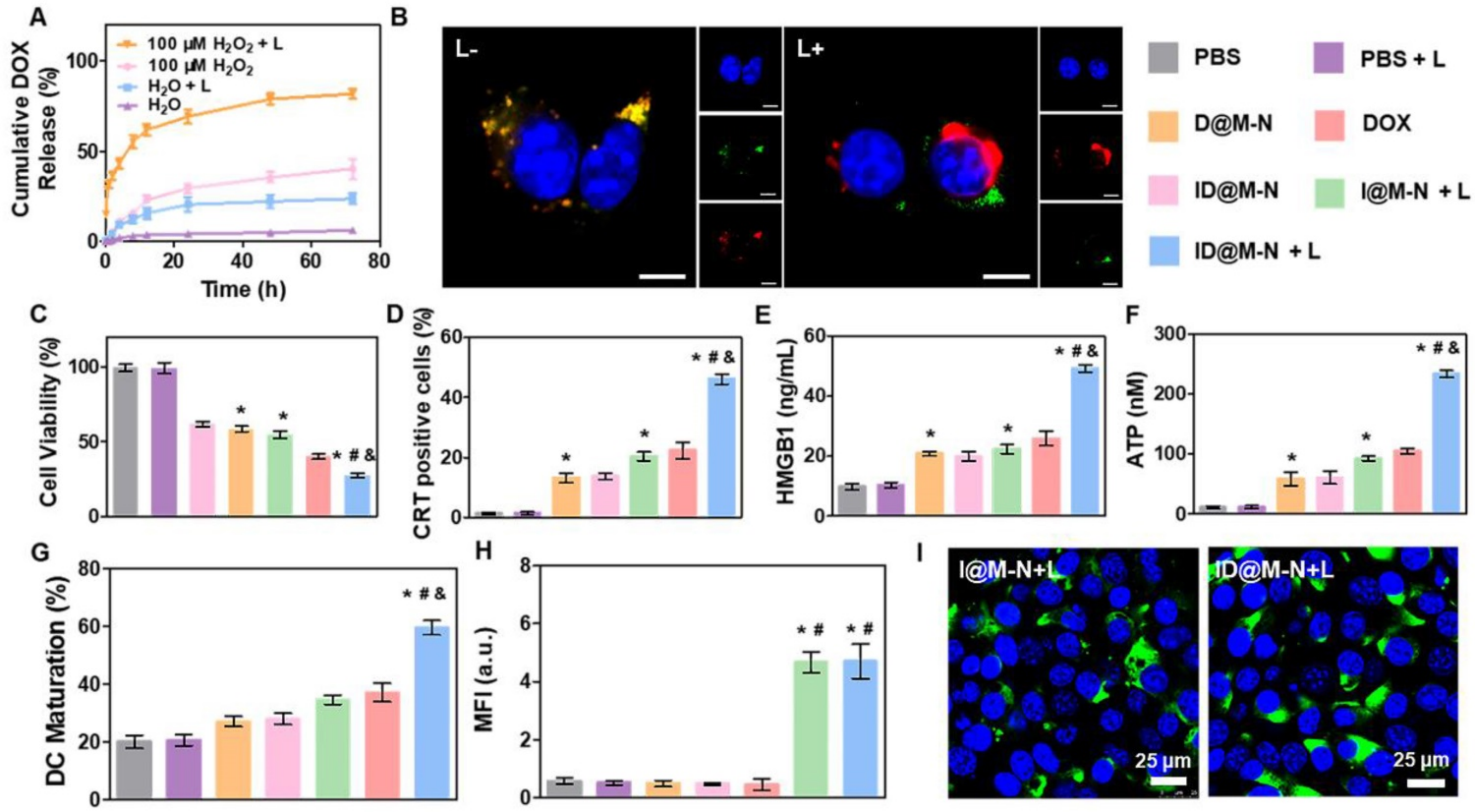
** Drug release and therapy* in vitro*. (A)** Drug release profiles under different conditions. **(B)** CLSM images of core-shell dissociation process of ID@M-N in 4T1 cells. Blue: nucleus, green: FITC-labeled shell layer of ID@M-N, red: DOX loading core of ID@M-N; scale bars: 10 µm.** (C)** *In vitro* cytotoxicity of different preparations (DOX: 3 µg mL^-1^, ID@M-N: 10.1 µg mL^-1^) on 4T1 cells after incubation for 1 day.** (D)** CRT-positive cells and **(E)** released HMGB1 and **(F)** ATP levels of 4T1 cells after 24 h exposure of different formulations. **(G)** Percentage of mature DCs (CD11c^+^CD80^+^CD86^+^) after co-incubation with different treated 4T1 cells. ^*, #, &^ P < 0.05 compared with the PBS (^*^), D@N-N (**^#^**), and I@M-N+L (**^&^**). **(H)** The mean fluorescence intensity (MFI) and **(I)** CLSM images of HSP70 expression on 4T1 cells after treated with different formulation. Blue: nucleus; green: HSP70 scale bars: 25 µm. ^*, #^ P < 0.05 compared with the PBS (^*^), D@N-N (**^#^**). All data are mean ± SD (n = 3).

To study the influence of light-driven transformation on drug release, the release profiles of DOX from ID@M-N were determined (Figure 3A). ID@M-N showed slow DOX release (<5% after 72 h) in media lacking light irradiation and H_2_O_2_. As a gatekeeper, the thermosensitive layer efficiently prevented DOX leakage. Approximately 40% of the DOX was released in 100 μM H_2_O_2_ solution after 72 h due to matrix degradation mediated the release (Figure 3A, Figure S11). In contrast, upon NIR light irradiation (808 nm, 2.5 W cm^-2^, 10 min), ID@M-N showed rapid and sustained DOX release (>80% after 48 h), suggesting that the irradiation boosted DOX was released under 100 μM H_2_O_2_ solution. The accelerated release mechanism might be explained by the NIR light-triggered dissociation and degradation of ID@M-N.

The influence of NIR light irradiation on DOX release was then evaluated in 4T1 cells. The intracellular fluorescence of DOX confirmed that ID@M-N could be efficiently taken up by 4T1 cells (Figure S12). ID@M-N exhibited rapid DOX release and facilitated DOX release into the cellular nucleus after NIR light irradiation. Moreover, intracellular ROS treated with ID@M-N upon NIR light irradiation was 19.2 times higher than the counterpart formulation in the dark (Figure S13). These results confirmed that ROS accelerated DOX release via dissociation and degradation of cellular ID@M-N. To investigate the dissociation process, FITC was used to label the thermosensitive layer shell of ID@M-N. Cellular FITC fluorescence co-localized with DOX in the dark (Figure 3B). In contrast, the co-localized fluorescence of FITC and DOX was separated after NIR light irradiation. These results revealed that NIR light-driven transformation of ID@M-N facilitated DOX release. We sought to investigate the influence of light-driven transformation of ID@M-N on 4T1 cell growth* in vitro*. ID@M-N exhibited lower cytotoxicity than D@M-N or free DOX because the thermosensitive layer prevented DOX release (Figure 3C, Figure S14). The cytotoxicity of ID@M-N + L against 4T1 breast cancer cells was 11.8 and 6.1 times higher than that of ID@M-N and I@M-N+L, respectively (Figure S14). Taken together, these findings demonstrate that NIR-triggered ID@M-N exhibits an efficient chemotherapeutic effect.

To investigate whether chemotherapy evoked the ICD effect and initiated an immune response, calreticulin (CRT) exposure, chromatin-binding protein high mobility group B1 (HMGB1), and adenosine triphosphate (ATP) release were first determined. After irradiation with NIR light, ID@M-N induced the highest percentage of CRT-positive cells (46.1%) (Figure 3D), and released the highest percentage of HMGB1 (Figure 3E) and ATP (Figure 3F). We also observed that ID@M-N with light irradiation greatly promoted *in vitro* DC maturation (59.6%) compared to any other single treatment (Figure 3G). Notably, the expression of heat shock protein 70 (HSP70), a common marker of immune activation, was measured (Figure 3H-I, Figure S15). Compared with other groups, both I@M-N and ID@M-N upon NIR light irradiation (0.25 W cm^-2^, 10 min) distinctly upregulated the expression of HSP70, indicating that the photothermal effect enhanced the immune response. Collectively, ID@M-N-based therapy sufficiently inhibited 4T1 cell growth and induced the ICD effect, which might trigger promising vaccine-like benefits for systematic anti-tumor therapy* in vivo*.

### *In vivo* Tumor Penetration and Biodistribution

**Figure 4:**
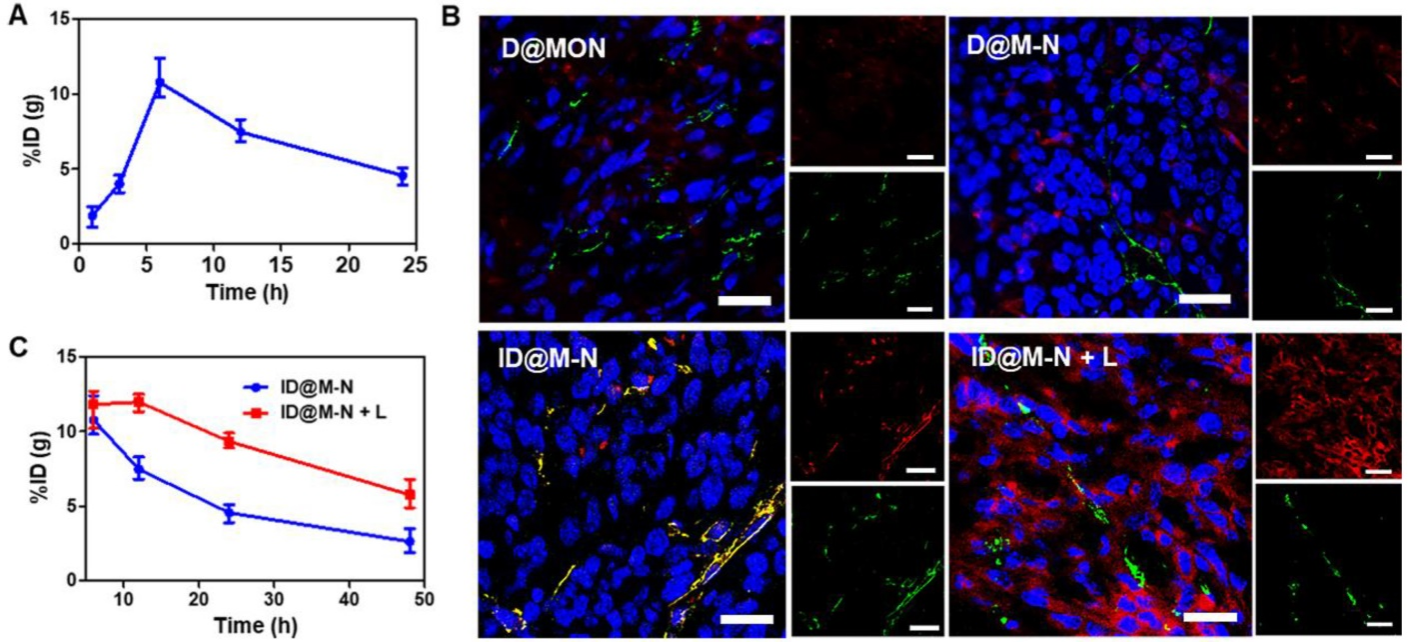
***In vivo* tumor penetration. (A)** Accumulation of ID@M-N at tumor site. **(B)** The tumor slice visualized tumor penetration of ID@M-N by CLSM imaging. Blue: nucleus; red: ID@M-N; green: blood vessels stained with anti-CD31-FITC; scale bars: 25 µm. **(C)** Accumulation of ID@M-N at tumor 6 h post-injection.

The biosafety profile of our nanosystems has been evaluated *in vivo*. First, hemolysis rate in mice of nanosystems has been evaluated as shown in Figure S16. The results showed no obvious hemolysis 44, 45. In order to measure the biological distribution of ID@M-N more accurately, we used ICP-MS to quantitatively analyze the *in vivo* distribution of Si element from ID@M-N nanoparticles. The results showed that nanoparticles mainly concentrated in liver and spleen as shown in Figure S17, suggesting that the endothelial system may be involved in drug metabolism and clearance 46. At the same time, the aggregation of nanoparticles in the tumor site increased with the extension of administration time, and reached the highest value 6 h after administration as shown in Figure 4A. The greatest accumulation of Si at the tumor site was observed at 6 h post-injection, which was defined as the optimal time for NIR light irradiation. The photothermal effect of ID@M-N was monitored using an infrared thermal imaging system (Figure S18). Compared with the PBS and free ICG groups, the temperature of ID@M-N increased to 43.2 °C for stimuli of the transformation.

We further investigated the NIR light-driven transformation of ID@M-N to enhance tumor penetration and accumulation* in vivo*. To prove this assumption, blood vessels were visualized by labeling with an anti-CD31 antibody. The fluorescence co-localization of the blood vessels (green) and ID@M-N (red) was observed using CLSM (Figure 4B). The fluorescence of DOX was weak in the D@M-N and D@MON groups, reflecting low accumulation in the tumor tissue. In contrast, the fluorescence intensity of ID@M-N with light irradiation suggested the highest accumulation in the tumor, which was 6.72-fold higher than that of ID@M-N (Figure 4B, Figure S19). The results supported that the use of light irradiation enhanced the accumulation of ID@M-N at the tumor site. In addition to enhancing accumulation, ID@M-N upon light irradiation was diffused to distant sites away from blood vessels, indicating deep tumor penetration. In contrast, ID@M-N without light stimuli was mainly localized around blood vessels. In facts, there is clear relationship that smaller size of nanoparticle facilitated enhanced permeability and retention effect 47. In addition, dense extracellular matrix in solid tumor will hinder nanoparticle's diffusion, resulting in poor penetration of lager nanoparticles 48. Importantly, as shown in Figure 4C, ID@M-N enhanced the retention at the tumor upon irradiation with NIR light. The increased blood perfusion by reducing the density of tumor structures and interstitial fluid pressure as a result of mild photothermal effects might further account for the increased penetration of nanomedicines 49-51. These results indicate that the light-driven transformable ID@M-N remarkably enhanced accumulation and deep tumor penetration.

### *In vivo* Anti-tumor Effects and Immunological Responses

**Figure 5:**
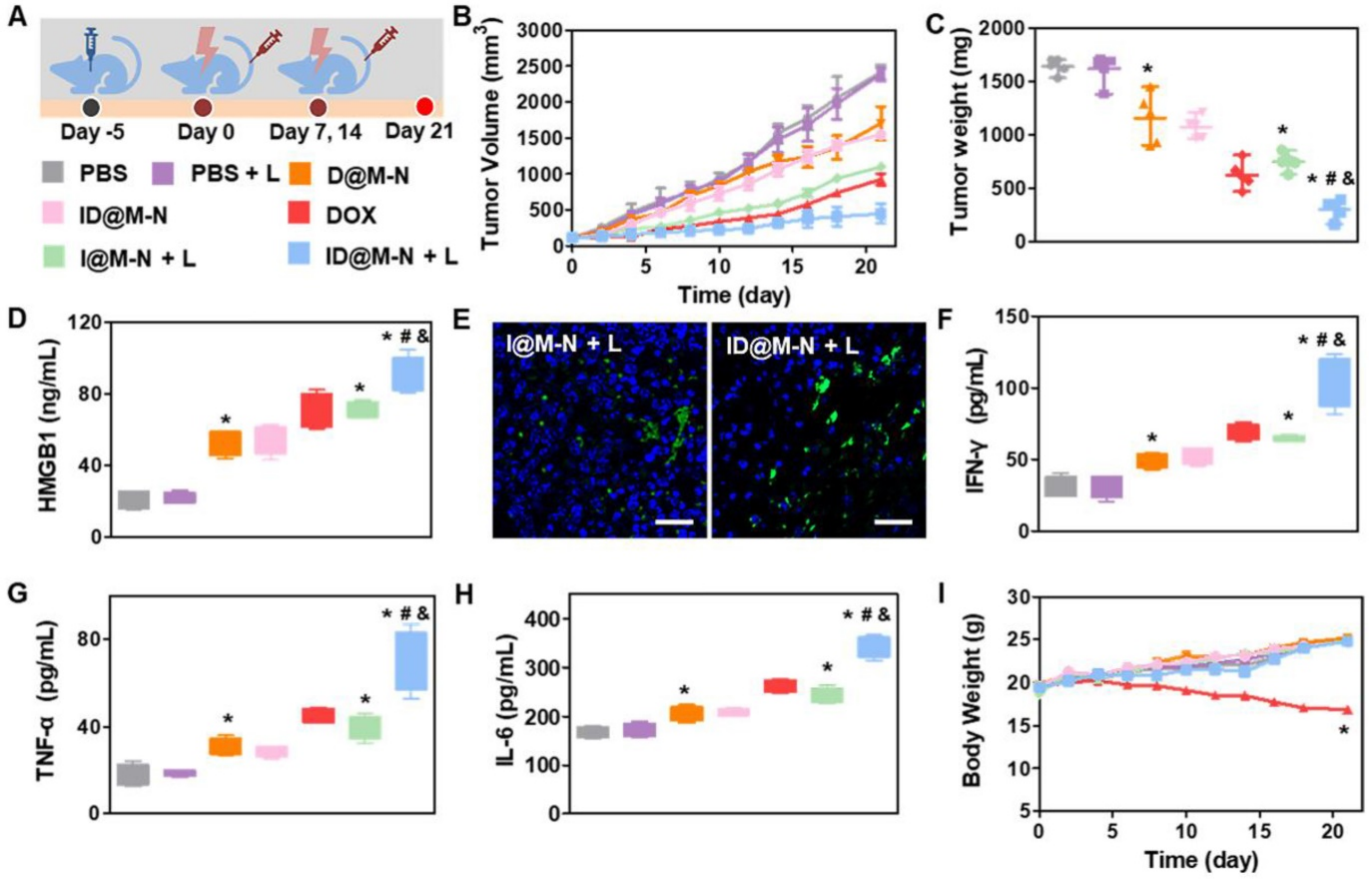
C**hemotherapy and anti-tumor immunologic response *in vivo*. (A)** Schematic treatment schedule. Mice were randomly divided into seven groups (n = 5): (1) PBS, (2) PBS+L, (3) D@M-N, (4) ID@M-N, (5) DOX, (6) I@M-N+L, (7) ID@M-N+L. **(B)** Tumor growth curves and** (C)** tumor weight. **(D)** Tumoral HMGB1 levels in each group. **(E)** CLSM images of HSP70 expression in 4T1 tumor. Blue: nucleus; green: HSP70; scale bar: 25 µm. **(F)** IFN-γ, **(G)** TNF-α, **(H)** IL-6 levels in serum after treatment. ^*, #, &^ P < 0.05 compared with the PBS (^*^), D@N-N (**^#^**), and I@M-N+L (**^&^**). **(I)** Body weight of mice in each group. All data are mean ± SD (n = 5). ^*^P < 0.05 compared with the PBS (^*^).

We then evaluated the therapeutic efficacy of ID@M-N-based chemotherapy in mice bearing 4T1 subcutaneous tumors (Figure 5A). The tumor-bearing mice were divided into seven groups: PBS, PBS + L, DOX, D@M-N, ID@M-N, I@M-N + L, and ID@M-N + L. Mice in the I@M-N + L, and ID@M-N + L groups were exposed to irradiation with NIR light (0.25 W cm^-2^, 10 min) at 6 h post-injection. Mice treated with DOX, D@M-N, and I@M-N + L exhibited partially delayed tumor growth (Figure 5B-C, Figure S20). Compared with DOX, D@M-N exhibited inferior therapy, which was attributed to the thermosensitive layer, hence preventing DOX release. As expected, ID@M-N + L groups showed the most outstanding anti-tumor performance, which benefited from sufficient tumor accumulation and deep penetration.

The immune responses elucidated by ID@M-N-based chemotherapy were further evaluated. The highest levels of HMGB1 were recorded in the ID@M-N + L group, revealing that chemotherapy induced ICD effects (Figure 5D). I@M-N + L and ID@M-N + L groups induced high expression levels of HSP70 in the tumor cells, indicating that mild photothermal effects facilitated the immune response (Figure 5E, Figure S21). The highest serum levels of IFN-γ, TNF-α and IL-6 in the ID@M-N + L group demonstrated a systemic immune response (Figure 5F-H). Furthermore, the safety profiles of the different treatments were evaluated. A decrease in body weight was observed in the DOX groups, revealing acute systemic side effects (Figure 5I). However, negligible systemic toxicity was recorded in the body weight, serum biochemistry, and histopathology of major organs, indicating that ID@M-N efficiently prevented leakage and reduced adverse effects (Figure S22-S23). These findings clearly demonstrated that ID@M-N-based efficient and safe chemotherapy evoked robust ICD effects and anti-tumor immune responses, which might achieve systematic anti-tumor performance when combined with a checkpoint inhibitor.

**Figure 6:**
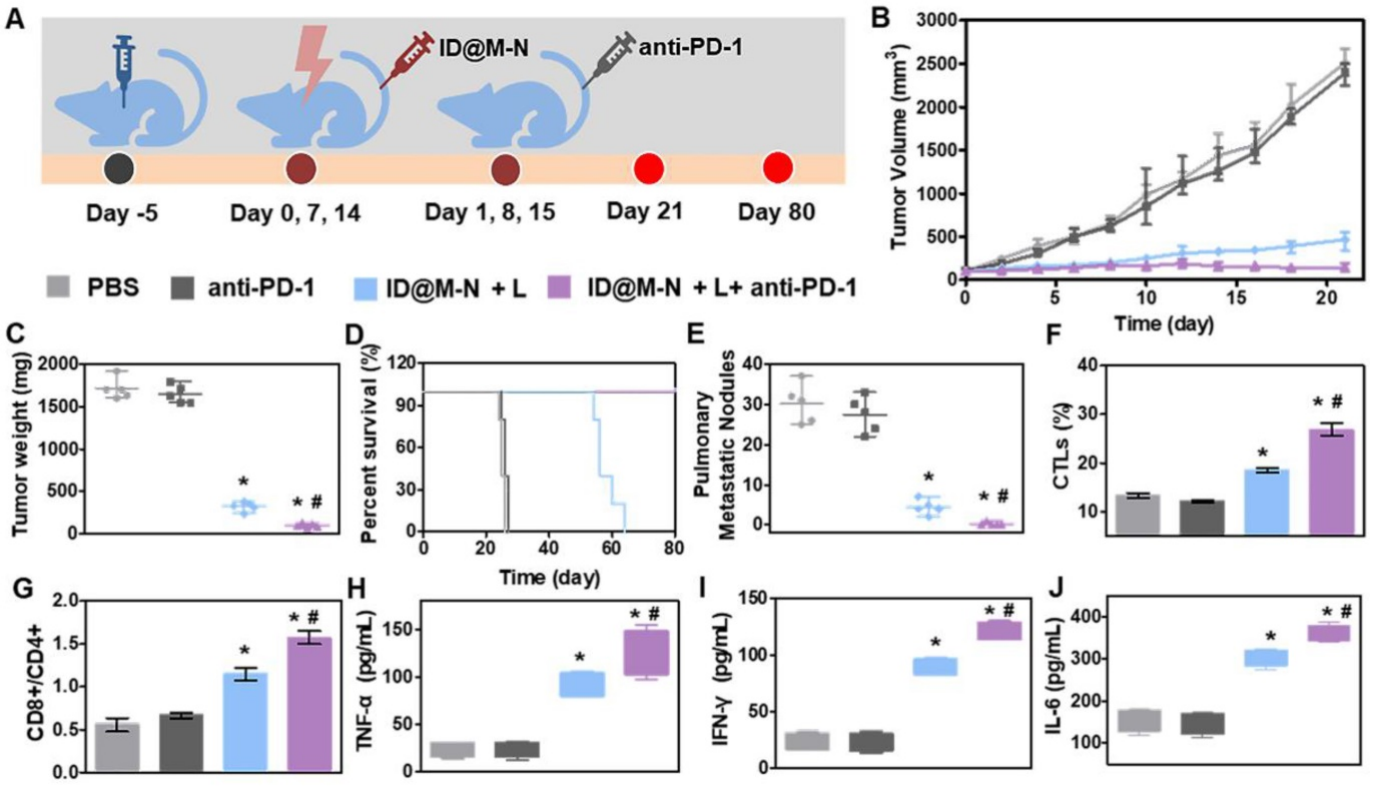
** Chemo-immunotherapy inhibited metastasis. (A)** Treatment schedule in 4T1 orthotopic mammary tumor model. Mice were randomly divided into four groups (n = 5): (1) PBS, (2) anti-PD-1, (3) ID@M-N+L, (4) ID@M-N+L+anti-PD-1. **(B)** Tumor growth curves, **(C)** tumor weight, **(D)** Survival time, and** (E)** number of pulmonary metastatic nodules in each group. **(F)** CTLs infiltration and **(G)** ratio of CD8^+^/CD4^+^ T cells in tumor tissue after 4 days of treatment. **(H)** TNF-α, **(I)** IFN-γ, **(J)** IL-6 levels in serum. All data are mean ± SD (n = 5). ^*, #^ P < 0.05 compared with the ID@M-N + L (**^#^**) and PBS (^*^).

Immune escape from tumors is one of key importance for metastasis at distant sites 52, 53. We investigated the systemic anti-tumor effects of ID@M-N+L with the aid of a PD-1 immune checkpoint inhibitor *in vivo* (Figure 6A). As shown in Figure 6B and C, immunotherapy alone (anti-PD-1 group) did not inhibit tumor growth. When combined with anti-PD-1, ID@M-N + L elicited stronger inhibition of tumor growth than ID@M-N + L. ID@M-N + L combined with immunotherapy further resulted in the highest apoptosis of the tumor cells, as revealed by hematoxylin-eosin (H&E) and terminal deoxynucleotidyl transferase dUTP nick-end labeling (TUNEL) staining of the tumor sections (Figure S24-S25). Notably, chemotherapy combined with immunotherapy not only remarkably prolonged the survival time of mice to 80 days (Figure 6D), but also prevented the development of pulmonary metastatic nodules (Figure 6E).

Intratumoral infiltration of immune cells was performed to further illustrate the systemic immune response. The population of CD8^+^/CD4^+^ T cells and the percentage of cytotoxic T lymphocytes (CTLs) significantly increased in the ID@M-N + L combined with anti-PD-1 group (Figure 6F-G). The proinflammatory cytokine production of TNF-α, IFN-γ, and IL-6 in tumor-bearing mouse sera were detected after treatment with various formulations. As expected, the higher levels of TNF-α, IFN-γ and IL-6 in the ID@M-N + L, combined with anti-PD-1 group confirmed that the combination of therapies efficiently evoked systemic anti-tumor immunity (Figure 6H-J). Systemic toxicity was evaluated after treatment. Compared with the normal group, the body weight, serum biochemistry, and hematological parameters showed no significant differences in the treated group (Figure S26-S28), demonstrating the inappreciable systemic toxicity of chemo-immunotherapy.

## Conclusion

In summary, we developed an NIR light-driven nanotransformer with deep tumor penetration for enhanced chemo-immunotherapy. ID@M-N underwent remarkable dual-transformation in size (from 115 to 20 nm) and surface charge (from negative to positive) under NIR light irradiation. When accumulated at the tumor site, ID@M-N achieved light-responsive dual-transformation by ROS and heat-mediated dissociation and degradation, which enhanced blood extravasation and deep tumor penetration capability. Consequently, such light-responsive transformable nanomedicine boosted robust ICD effects and elicited systemic anti-tumor immunity. When combined with anti-PD-1, ID@M-N-based chemotherapy evoked systemic anti-tumor immunity to significantly suppress primary tumor growth and pulmonary metastasis of breast cancer. This study offers a new NIR light-responsive nanomedicine with enhanced accumulation and deep tumor penetration for efficient and safe chemo-immunotherapy.

## Supplementary Material

Supplementary methods, figures and table.

## Abbreviations

NIR: near infrared
ROS: reactive oxygen species
ICG: indocyanine green
DOX: doxorubicin
PD-1: programmed cell death protein-1
ICD: immunogenic cell death
NIPAM: *N*-isopropyl acrylamide
MONs: mesoporous organosilica nanoparticles
MBA: *N, N'*-methylenebisacrylamide
APS: ammonium persulphate
PEI: polyethyleneimine
PTT: photothermal therapy
LC: loading capacity
TGA: Thermogravimetric analysis
LCST: lower critical solution temperature
MTT: methyl thiazolyl tetrazolium
FTIR: Fourier transform infrared
DLS: dynamic light scattering
TEM: transmission electron microscopy
SEM: scanning electron microscopy
UV-Vis: ultraviolet visible
H&E: hematoxylin-eosin
TUNEL: terminal deoxynucleotidyl transferase dUTP nick end labeling
