# Supplementary material for: A light-driven dual-nanotransformer with deep tumor penetration for efficient chemo-immunotherapy
Source: Theranostics. 2022 Jan 24;12(4):1756–68. doi: 10.7150/thno.68756 (PMC8825592; doi:10.7150/thno.68756)
Supplement: Supplementary file 1 — Supplementary methods, figures and table. [file thnov12p1756s1.pdf]

Supporting Information

A Light-Driven Dual-Nanotransformer with Deep Tumor Penetration for Efficient Chemo-Immunotherapy

Jiahui Peng[1,2], Fangman Chen[1,2,], Yulu Liu[1,2], Fan Zhang[2], Lei Cao[1,2], Qiannan You[1,2], Dian Yang[1,2], Zhimin Chang[2], Mingfeng Ge[2], Li Li[2], Zheng Wang[6], Qian Mei[2], Dan Shao[3,4,5,*], Meiwan Chen[7] and Wen-fei Dong[1,2,*]*

[1] School of Biomedical Engineering (Suzhou), Division of Life Sciences and Medicine, University of Science and Technology of China, 96 Jinzhai Road, Hefei 230026, China

[2] CAS Key Laboratory of Bio Medical Diagnostics, Suzhou Institute of Biomedical Engineering and Technology Chinese Academy of Sciences, 88 Keling Road, Suzhou 215163, China

[3] Institutes of Life Sciences, School of Medicine, South China University of Technology, Guangzhou, Guangdong 510006, China

[4] School of Biomedical Sciences and Engineering, South China University of Technology, Guangzhou, Guangdong 510006, China

[5] National Engineering Research Center for Tissue Restoration and Reconstruction, South China University of Technology, Guangzhou, Guangdong 510006, China

[6] CAS Key Laboratory of Nano-Bio Interface, Suzhou Institute of Nano-Tech and Nano-Bionics, Chinese Academy of Sciences, 398 Ruoshui Road, Suzhou 215123, China

[7] State Key Laboratory of Quality Research in Chinese Medicine, Institute of Chinese Medical Sciences, University of Macau, Macau, China

*Corresponding author: Fangman Chen, E-mail: chenfangman@hotmail.com. Dan Shao, Prof., E-mail:stanauagate@outlook.com. Wen-fei Dong, Prof., E-mail: wenfeidong@sibet.ac.cn.

Cytotoxicity assay

4T1 cells were seeded into a 96-well plate at a density of 5×10^3 cells per well. After 24 h of culture, cells were treated with fresh medium containing M-N, DOX, D@M-N, ID@M-N, and I@M-N (equal to DOX at varying concentrations: 0.1, 0.2, 0.5, 1, 2, 3, 4, and 5 μg mL^{-1}), and the untreated cells were served as a control group. After 2 h of incubation, cells were either irradiated or were not irradiated with NIR light (0.25 W cm^{-2}) for 10 min and then cultured for another 22 h. WST-1 kit was used to investigate the toxicities of the different formulations. Briefly, WST-1 reagent (20 μL) was added to each well when the incubation was completed, and the absorbance at a wavelength of 450 nm was measured.

Immunogenic cell death (ICD) and DC maturation in vitro

4T1 cells were seeded into 24-well plates (10×10^4 cells per well). After 24 h of culture at 37°C, the cells were treated as follows for 24 h: M-N, DOX, D@M-N, I@M-N+NIR, ID@M-N, ID@M-N+NIR (0.25 W cm^{-2}, 10 min). For the detection of HMGB1 and ATP secretion, cell culture supernatants were collected and analyzed using an ELISA kit. For HSP70 expression analysis, cells were stained with anti-HSP70 antibody and DAPI for observation by CLSM. For calreticulin (CRT) expression, cells were collected and stained with an anti-CRT antibody for FACS analysis. To detect dendritic cell maturation, DCs from mouse bone marrow were co-incubated with treated 4T1 cells for 24 h. DCs were collected and stained with anti-CD11c-FITC, anti-CD80-PE, and anti-CD86-APC for FACS analysis.

Hemolysis assay

Red blood cells isolated from fresh blood of Balb/C mice were diluted with PBS to the concentration of 5×10^7 cells mL^{-1}. 200 μL of diluted red blood cells suspension was mixed with different formulations: D@M-N, I@M-N, and ID@M-N ($C_{M\text{-}N}$: 0.2, 0.4, 0.6, 0.8, 1 mg mL^{-1}). Then the mixture was incubated at 37 ° C for 1 h, with PBS as negative control and 1% Triton as positive control. After centrifugation (3000 rpm, 10 min), the absorbance value of supernatant solution at 545 nm was detected. Hemolysis rate was calculated according to the following formula:

$$\text{Hemolysis\%}=\frac{A-A_n}{A_p-A_n}\times 100\% \quad (1)$$

(A, A_n and A_p are the absorbance of the experiment group, the negative group, and the positive group.)

Biodistribution, photothermal effect, and tumor penetration in vivo

All animal experimental protocols were approved by the ethical committee and in strict accordance with the recommendations of the Suzhou Institute of Biomedical Engineering and Technology, Chinese Academy of Sciences Laboratory Animal Center. The 4T1 tumor model was established in BALB/c mice with a subcutaneous injection of 5×10^5 4T1 cells. To determine the optimal time of irradiation with light in vivo, 4T1 tumor-bearing mice were intravenously injected with ID@M-N (NPs: 5 mg kg^{-1}). At predetermined time intervals (1, 3, 6, 12, and 24 h), tumors and major organs including the heart, liver, spleen, lung, and kidney were harvested for the measurement of silicon (Si) content by ICP-OES.

The photothermal effect was evaluated when the tumor volume reached approximately 200 mm^3 by intravenously injecting PBS, free ICG, and ID@M-N with an equivalent ICG concentration of 0.52 mg kg^{-1} into the tumor-bearing mice (200 μL per mouse). After 6 h, mice were exposed to the 808 nm laser (0.25 W cm^{-2}) for 10 min. The photothermal effect was recorded using a thermal imaging camera (FOTRIC, China).

To investigate the deep penetration of ID@MON, 4T1 tumor-bearing mice were intravenously injected with D@MON, D@M-N, ID@M-N, and ID@M-N+L (1 mg kg^{-1} DOX, 0.52 mg kg^{-1} ICG). The mice were exposed to irradiation (0.25 W cm^{-2}, 10 min) after 6 h. The tumors were harvested after 2 h. Tumor sections were stained with anti-CD31-FITC and DAPI.

Chemotherapy and immunological responses in vivo

When the tumor volume reached approximately 100 mm^3, 4T1 tumor-bearing mice were randomly divided into seven groups (n=5), and mice were administered with PBS, PBS+L, DOX, D@M-N, I@M-N+L, ID@M-N, ID@M-N+L (1 mg kg^{-1} DOX, 0.52 mg kg^{-1} ICG) once every 7 days. The 4T1 tumor-bearing mice in the laser group were irradiated for 10 min (0.25 W cm^{-2}) at 6 h post administration. Tumor volume (tumor volume = 0.5 × tumor length×tumor width^2) and body weight were measured every 2 days. Serum TNF-α, IFN-γ, and IL-6 levels were analyzed at fourth days. The level of HMGB1 in the tumor tissue was detected using ELISA. HSP70 expression in tumors was detected by immunofluorescence staining. After treatment, all mice were sacrificed, and their main organs including the heart, liver, spleen, lung, and kidney were collected for hematoxylin and eosin (H&E) staining. Biochemical parameters, including AST, ALT, BUN, CREA, and creatine kinase (CK), were analyzed automatically using Coulter LX2D instrumentation (Beckman, Brea, CA).

Chemotherapy combined with immunotherapy inhibited metastasis

4T1 tumor-bearing mice were randomly divided into four groups (n=5): PBS, anti-PD-1, ID@M-N+L, ID@M-N+L+anti-PD1 (1 mg kg^{-1} DOX, 0.52 mg kg^{-1} ICG, anti-PD1 100 μg per mouse). The 4T1 tumor-bearing mice in the laser group were irradiated for 10 min (0.25 W cm^{-2}) at 6 h post administration. Tumor volume and body weight were measured every two days. In addition, TdT-mediated dUTP nick-end labeling (TUNEL) staining was used to evaluate tumor apoptosis. Serum samples of mice were collected and analyzed for the levels of TNF-α, IFN-γ, and IL-6 by ELISA after the above administration. To assess cytotoxic T lymphocytes (CTLs, $CD3^+CD4^-CD8^+$), $CD8^+$ T cells ($CD3^+CD4^-CD8^-$), $CD4^+$ T cells ($CD3^+CD4^+CD8^-$), and lymphocytes from the tumor tissue were isolated and stained with anti-CD3-FITC, anti-CD4-PE, and anti-CD8-APC, and the cells were analyzed by FACS. In separate experiments, 4T1 tumor-bearing mice were treated as described above (n=5), and the survival time of each mouse was recorded. All mice were sacrificed after treatment, and the main organs, including the heart, liver, spleen, lung, and kidney were collected for H&E staining. Biochemical parameters, including AST, ALT, BUN, CREA, and CK, were analyzed automatically using Coulter LX2D instrumentation (Beckman, Brea, CA).

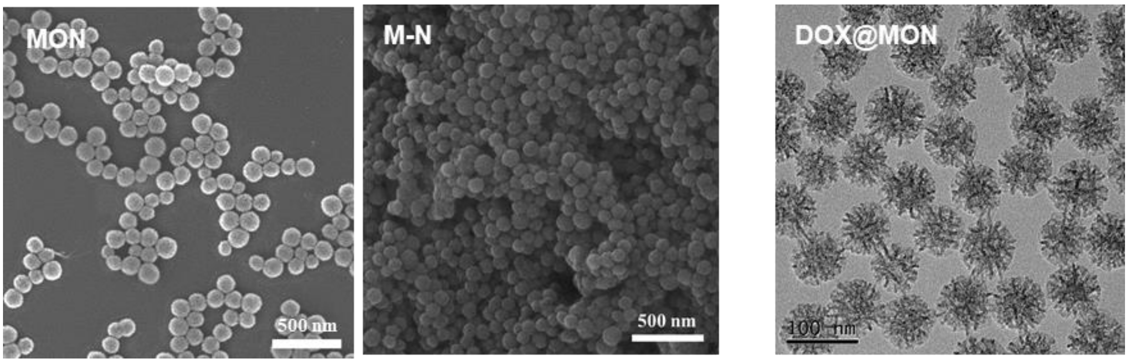

Figure S1. SEM image of MON and M-N, and TEM image of D@MON.

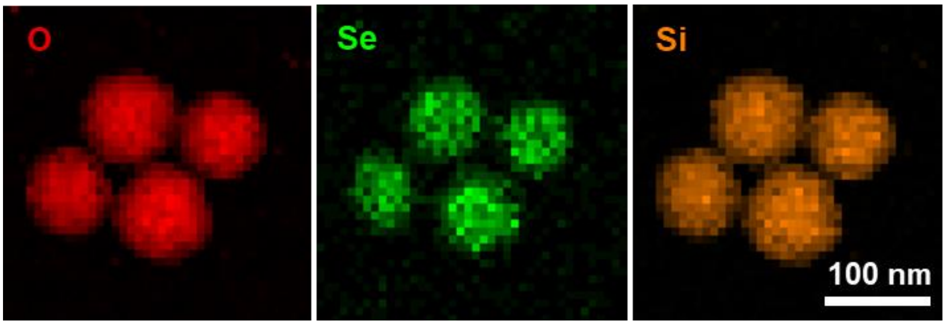

Figure S2. Energy-dispersive X-ray spectroscopy (EDS) mapping of MON.

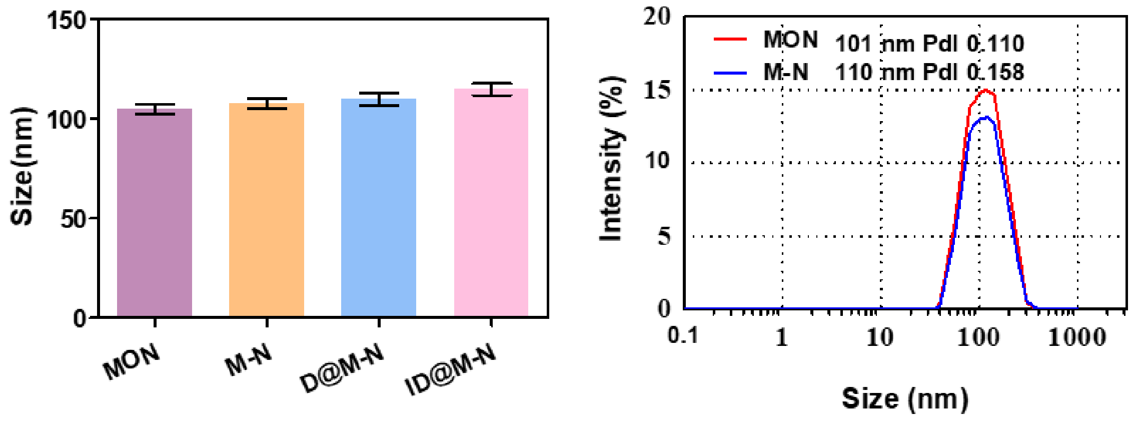

Figure S3. Particle size and size distribution of different formulations.

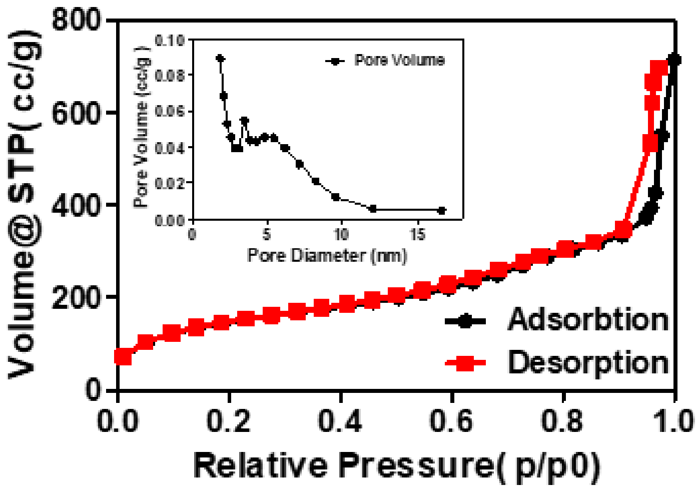

Figure S4. Nitrogen adsorption-desorption isotherms and pore size distribution of MON.

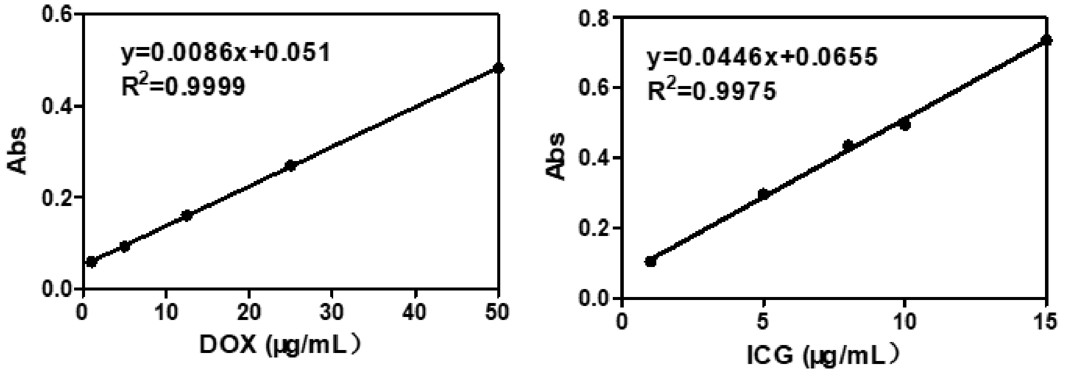

Figure S5. Standard concentration curve of DOX and ICG based on UV-visible spectra.

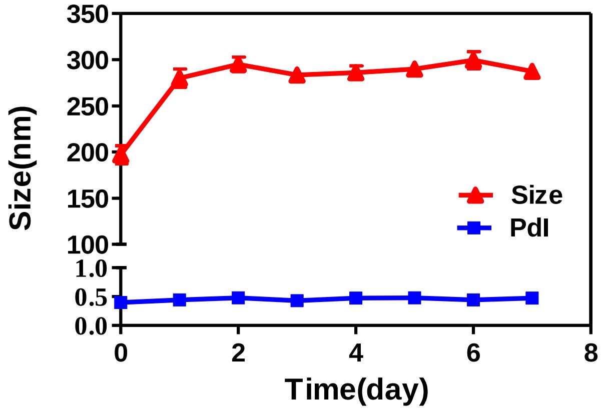

Figure S6. Colloidal stability of ID@M-N in 50% FBS-containing culture media.

.

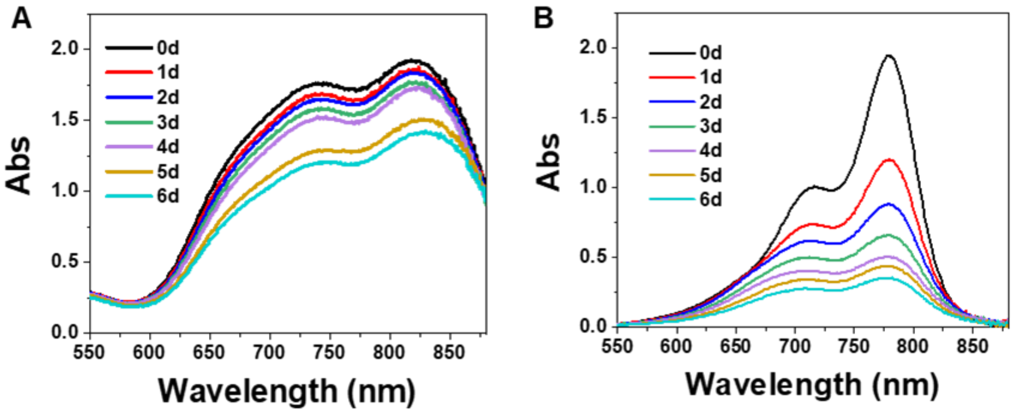

Figure S7. Stability of **(A)** ID@M-N and **(B)** free ICG based on UV-vis spectra.

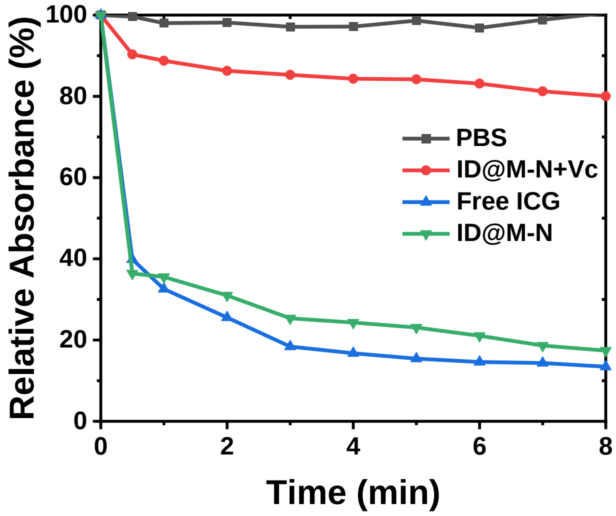

Figure S8. Relative absorbance of PBS, ID@M-N+Vc, ID@M-N and free ICG mixed with DPBF after irradiation with 808 nm NIR (0.25 W cm^{-2}).

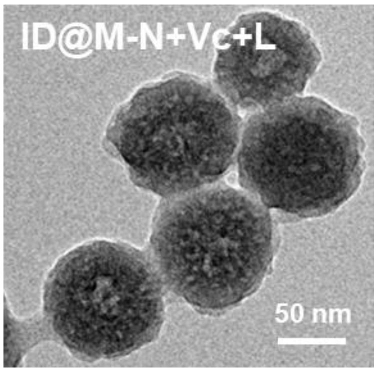

Figure S9. Degradation of ID@M-N+Vc upon light irradiation (0.25 W cm^{-2}, 10 min).

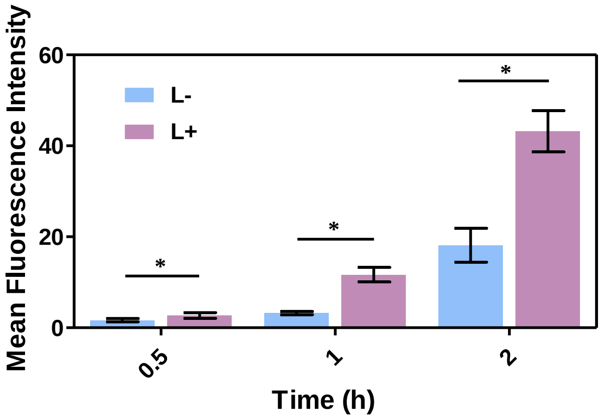

Figure S10. Mean fluorescence intensity of 4T1 cells uptake of ID@M-N with or without NIR light pretreated (10 min, 0.25 W cm^{-2}).

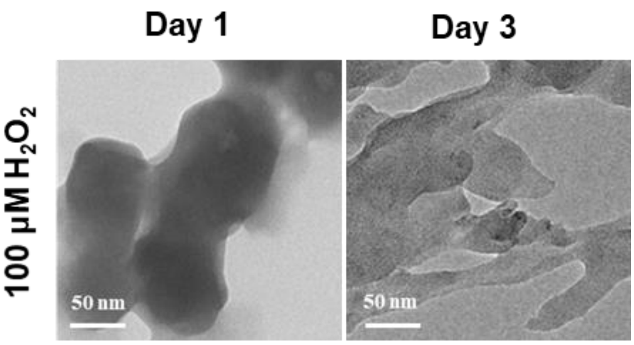

Figure S11. Degradation of MON under 100 μM H_2O_2 solution.

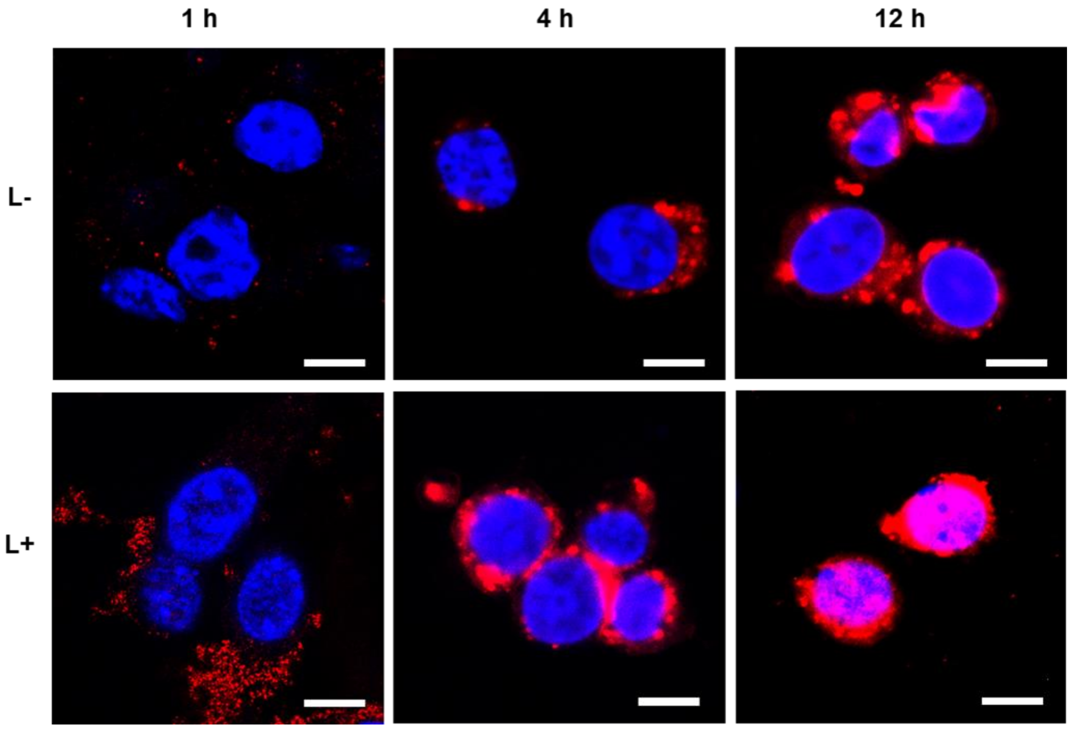

Figure S12. CLSM images of 4T1 cells incubated with ID@M-N at 1, 4 and 12 h after light irradiation (0.25 W cm^{-2}, 10 min). Blue: nucleus; red: DOX; scale bars: 10 μm.

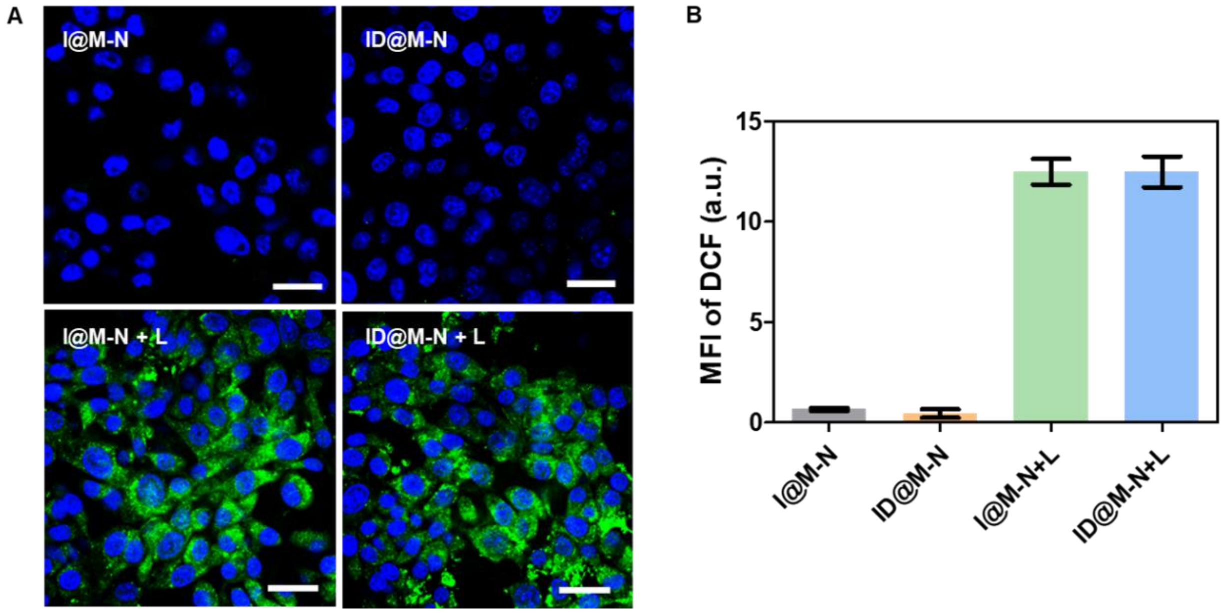

Figure S13. (A) CLSM images and **(B)** mean fluorescence intensity of DCFH-DA in 4T1 cells after treatment with I@M-N and ID@M-N. Blue: nucleus; green: DCFH-DA; scale bars: 50 μm.

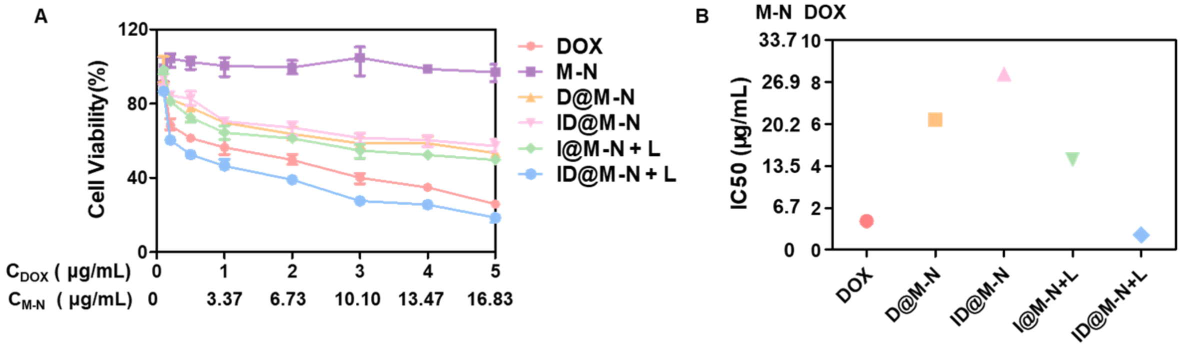

Figure S14. *In vitro* cytotoxicity IC50 value of different preparations against 4T1 cells.

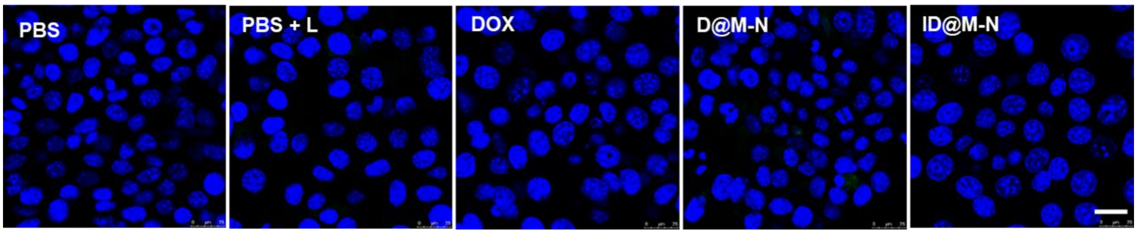

Figure S15. CLSM images of HSP70 expression in 4T1 cells treated with different formulations upon light irradiation (0.25 W cm^{-2}, 10 min). Blue: nucleus; green: HSP70; scale bars: 25 μm.

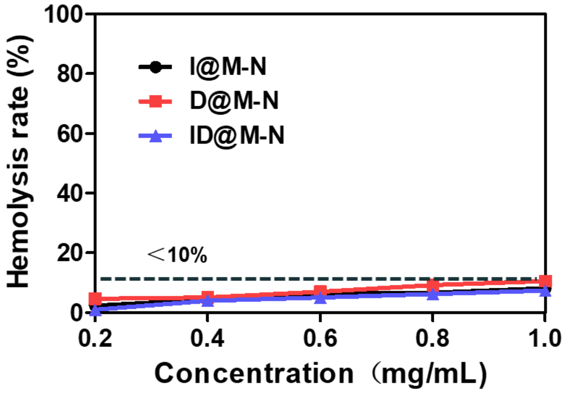

Figure S16. Hemolysis rate of different nanoparticles with different concentrations.

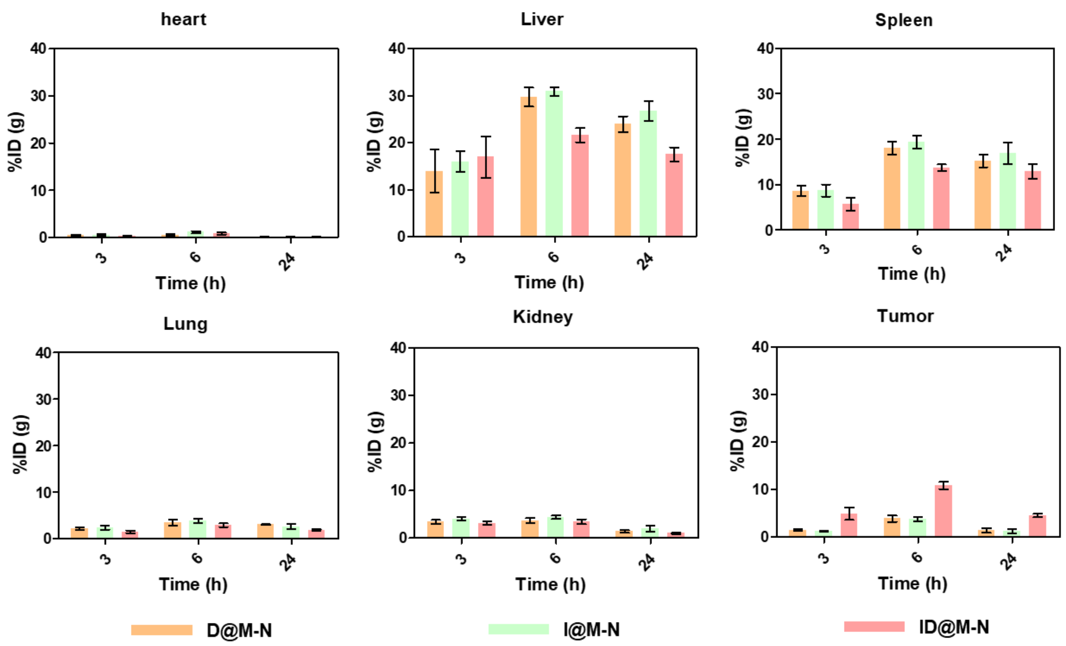

Figure S17. Biodistribution of D@M-N, I@M-N and ID@M-N in 4T1 tumor-bearing mice post injection at different time points.

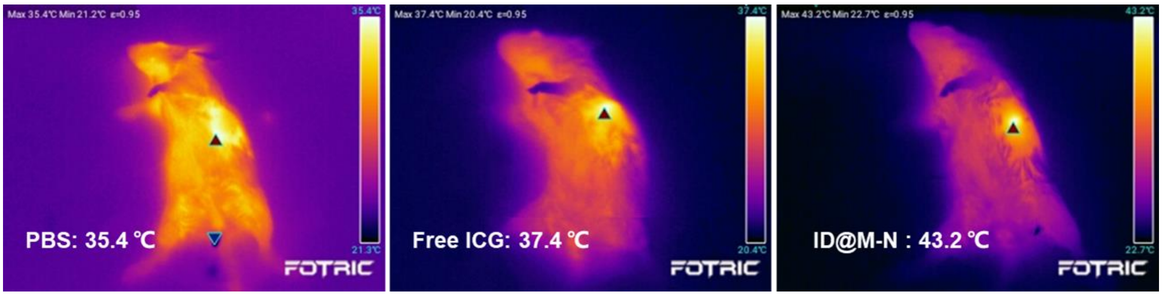

Figure S18. *In vivo* photothermal effects of PBS, free ICG and ID@M-N upon light irradiation (0.25 W cm^{-2}, 10 min).

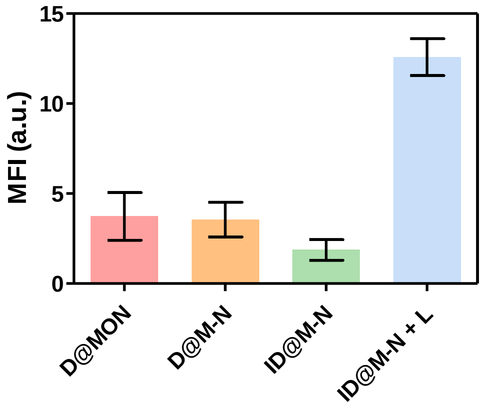

Figure S19. MFI of DOX in CLSM images of tumor sections.

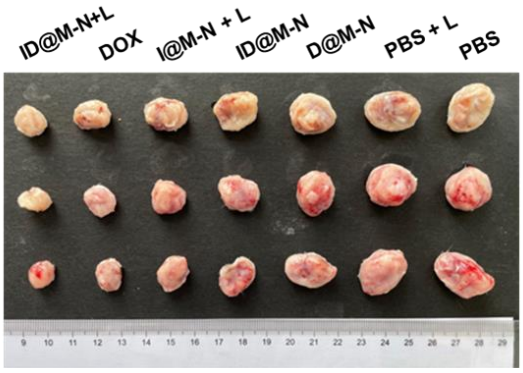

Figure S20. Tumor photographs after different treatments.

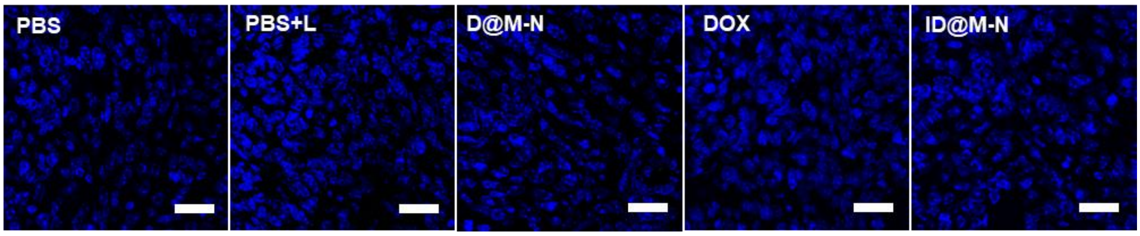

Figure S21. CLSM images (blue: nucleus; green: HSP70) of HSP70 expression in 4T1 tumor-bearing mice after different treatment; scale bars: 25 μm.

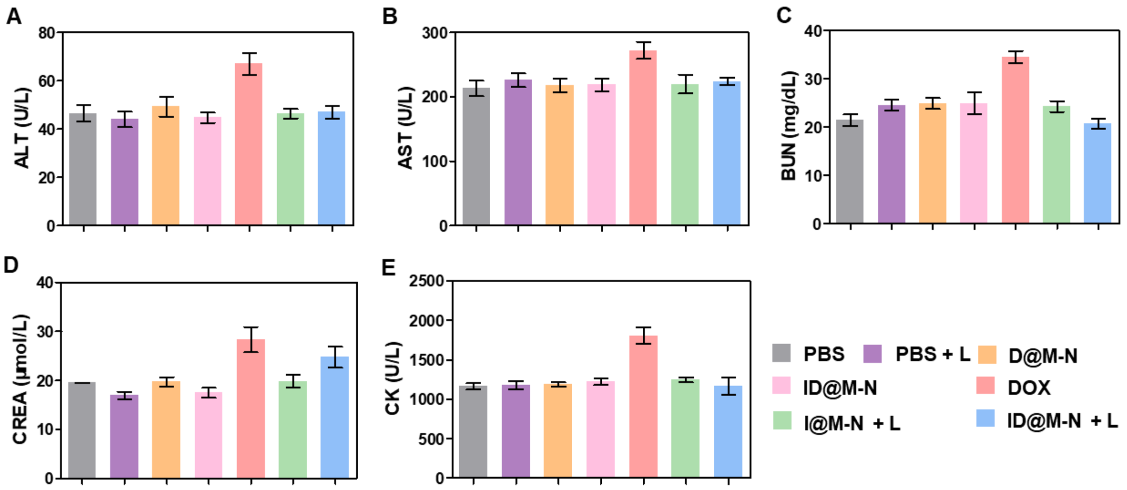

Figure S22. Biochemical parameters including **(A)** alanine aminotransferase (ALT), **(B)** aspartate aminotransferase (AST), **(C)** blood urea nitrogen (BUN), **(D)** creatinine (CREA), and **(E)** creatine kinase (CK) of 4T1 tumor-bearing mice after 21-day of treatment. Data represent the mean ± SD (n=5).

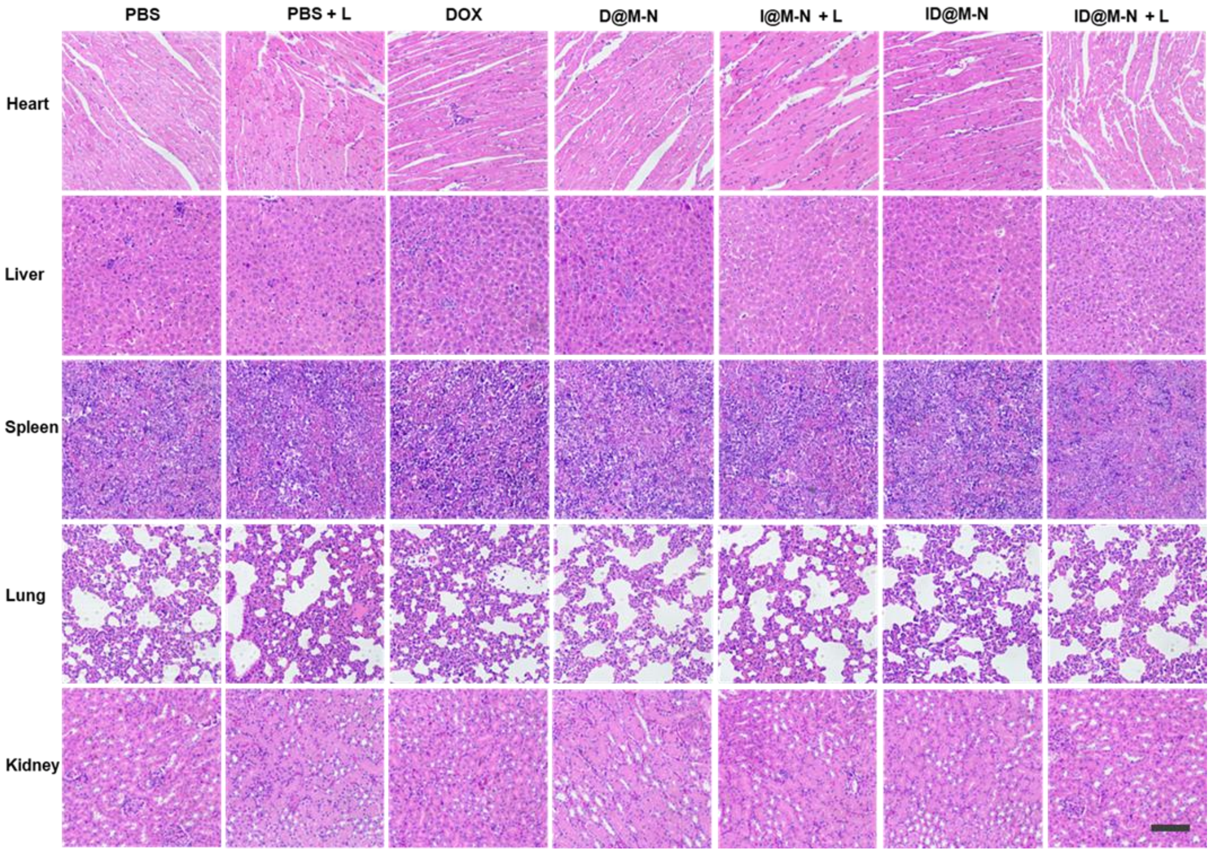

Figure S23. Histological evaluation from the major organs including the heart, liver, spleen, lung and kidney of 4T1 tumor-bearing mice after 21-day of treatment; scale bars: 100 μm.

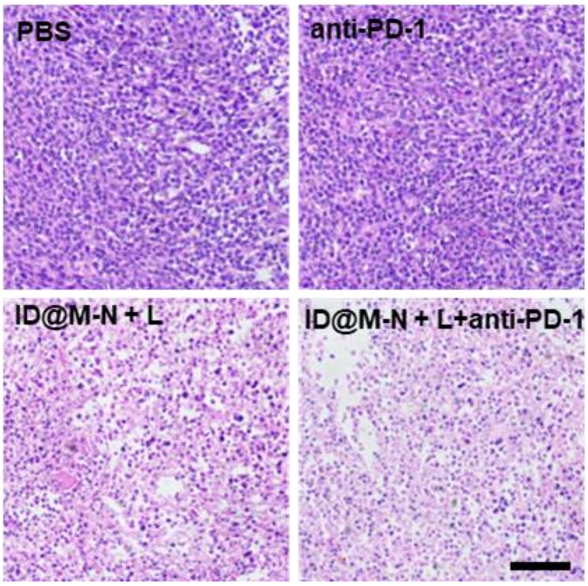

Figure S24. H&E images of tumors; scale bars: 100 μm.

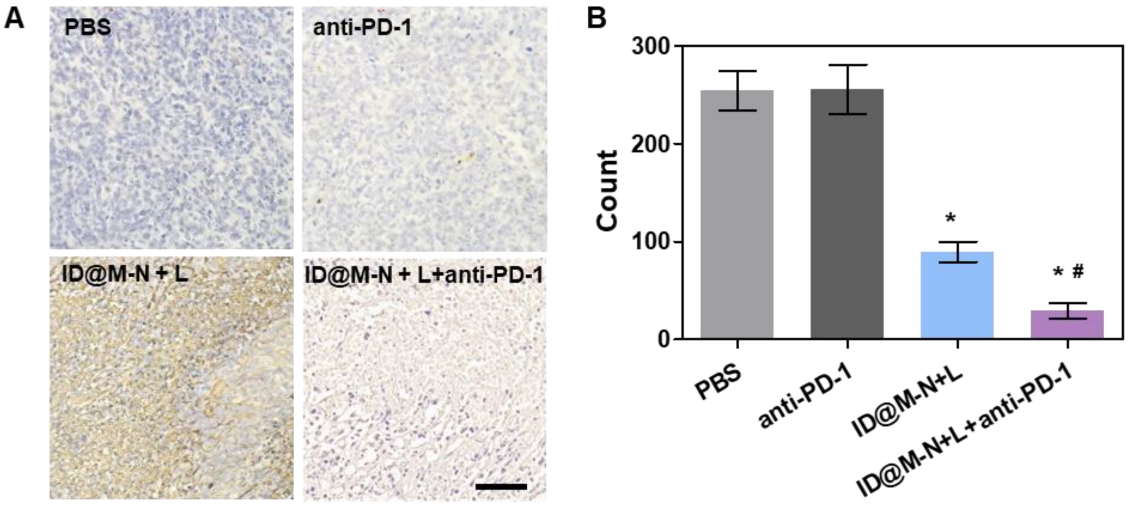

Figure S25. (**A**) TUNEL images and **(B)** positive cell count of tumors; scale bars: 100 μm. All data are mean ± SD (n=5). *, # P＜0.05 compared with the ID@M-N + L (#) and PBS (*).

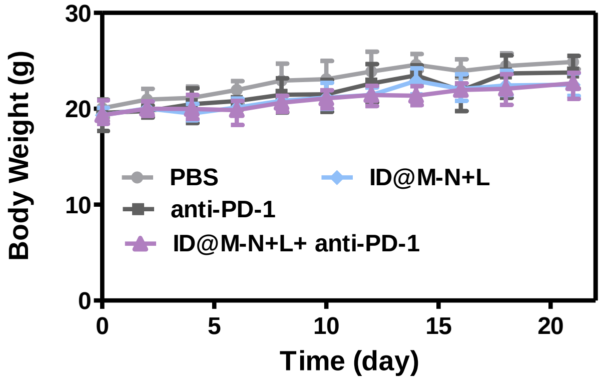

Figure 26. Body weight of mice after treatments.

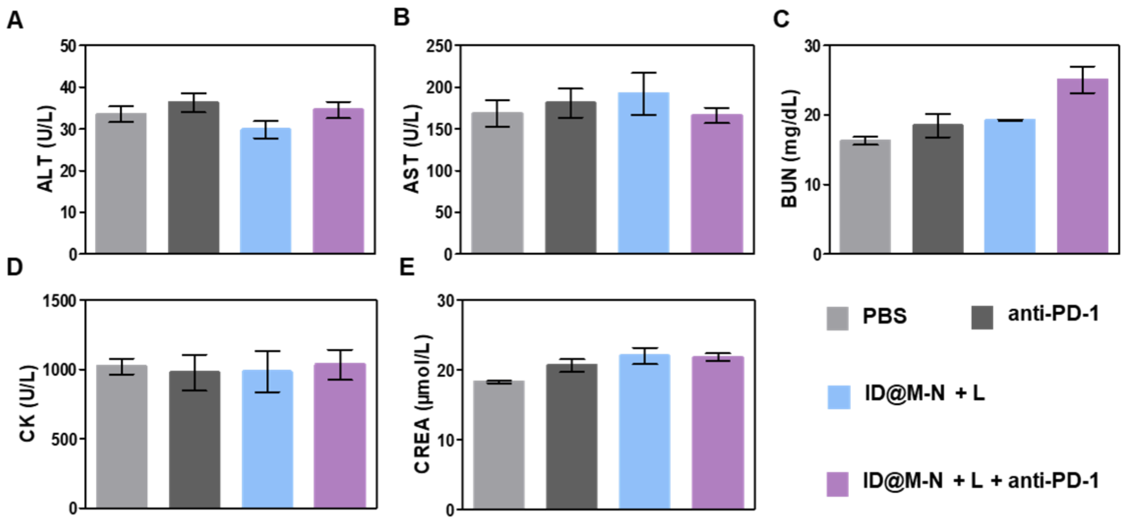

Figure S27. Biochemical parameters including **(A)** alanine aminotransferase (ALT), **(B)** aspartate aminotransferase (AST), **(C)** blood urea nitrogen (BUN), **(D)** creatine kinase (CK) and **(E)** creatinine (CR) of 4T1 tumor-bearing mice after 21-day of treatment. Data represent the mean ± SD (n=5).

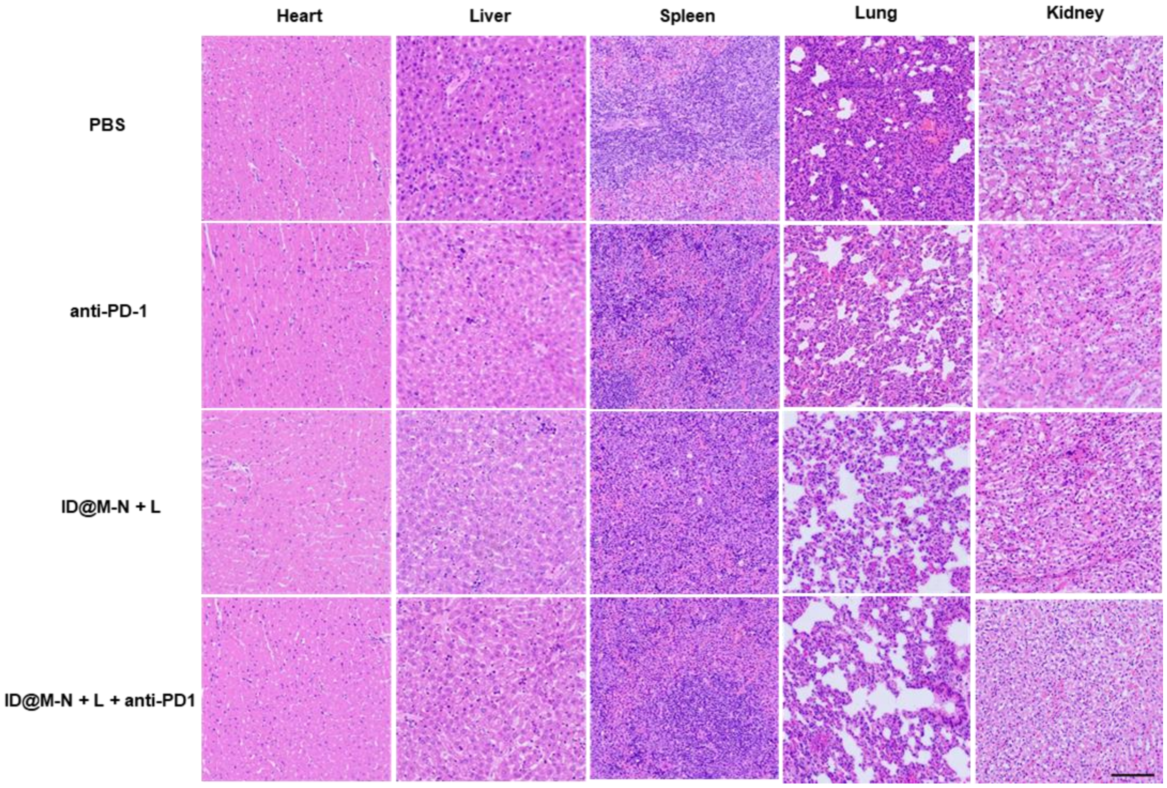

Figure 28. Histological evaluation of major organs including the heart, liver, spleen, lung and kidney of 4T1 tumor-bearing mice after treatment; scale bars: 100 μm.

Table S1. BET surface, pore volume and pore size of MON and MON-NIPAM.

	BET Surface ($m^2 g^{-1}$)	Pore Volume ($cm^3 g^{-1}$)	Pore Size(nm)
MON	541.066	1.012	8.164
M-N	78.69	0.7744	2.69
